# RNA-dependent RNA polymerases regulate ascospore discharge through the exonic-sRNA-mediated RNAi pathway

**DOI:** 10.1128/mbio.00377-24

**Published:** 2024-05-16

**Authors:** Wenping Zeng, Jing Lin, Jiatao Xie, Yanping Fu, Yang Lin, Tao Chen, Bo Li, Xiao Yu, Weidong Chen, Daohong Jiang, Jiasen Cheng

**Affiliations:** 1 Key Laboratory of Environment Change and Resources Use in Beibu Gulf, Ministry of Education, Nanning Normal University, Nanning, China; 2 National Key Laboratory of Agricultural Microbiology, Huazhong Agricultural University, Wuhan, China; 3 The Provincial Key Lab of Plant Pathology of Hubei Province, College of Plant Science and Technology, Huazhong Agricultural University, Wuhan, China; 4 USA Department of Agriculture, Agricultural Research Service, Washington State University, Pullman, Washington, USA; Cornell University, Ithaca, New York, USA

**Keywords:** sexual development, RNA-dependent RNA polymerase, exonic small RNAs, *Fusarium graminearum*

## Abstract

Ascospores, forcibly released into the air from perithecia, are the primary inoculum for Fusarium head blight. In *Fusarium graminearum*, the biological functions of four RNA-dependent RNA polymerases (RdRPs) (*Fgrdrp1–4*) have been reported, but their regulatory mechanisms are poorly understood and the function of *Fgrdrp5* is still unknown. In this study, we found that in addition to *Fgrdrp1* and *Fgrdrp2*, *Fgrdrp5* also plays an important role in ascospore discharge, and they all participate in the generation of turgor pressure in a polyol-dependent manner. Moreover, these three genes all affect the maturation of ascospores. Deep sequencing and co-analysis of small RNA and mRNA certified that *Fgrdrp1*, *Fgrdrp2*, and *Fgrdrp5* partly share their functions in the biogenesis and accumulation of exonic small interference RNA (ex-siRNA), and these three RdRPs negatively regulate the expression levels of ex-siRNA corresponding genes, including certain genes associated with ascospore development or discharge. Furthermore, the differentially expressed genes of deletion mutants, those involved in lipid and sugar metabolism or transport as well as sexual development-related transcription factors, may also contribute to the defects in ascospore maturation or ascospore discharge. In conclusion, our study suggested that the components of the *dicer*-dependent ex-siRNA-mediated RNA interference pathway include at least *Fgrdrp1*, *Fgrdrp2*, and *Fgrdrp5*.

**IMPORTANCE:**

We found that in addition to *Fgrdrp1* and *Fgrdrp2*, *Fgrdrp5* also plays important roles in ascospore maturation and ascospore discharge of *Fusarium graminearum*. These three RNA-dependent RNA polymerases participate in the biogenesis and accumulation of exonic small interference RNA and then regulate ascospore discharge.

## INTRODUCTION

*Fusarium graminearum*, a filamentous ascomycete, mainly causes Fusarium head blight (FHB) and Gibberella ear rot in small-grain cereals (1, 2). Reproduction of this pathogen mainly relies on sexual spores (ascospores) and asexual spores (conidia). Ascospores serve as the primary inoculum for FHB outbreaks as they are forcibly emitted into the air from the perithecia (fruiting bodies) and spread over long distances (3). Moreover, the production of perithecia during sexual reproduction is essential for the overwintering of *F. graminearum* (4). Therefore, understanding the molecular mechanisms of sexual development and ascospore discharge is important for the prevention and control of head blight.

As a homothallic ascomycete, sexual reproduction in *F. graminearum* can be triggered without mating, and each ascus must undergo mitosis and cytokinesis to assemble eight mature ascospores with four cells and four nuclei (5). After the ascospore matures, osmolytes accumulate in the asci, generating turgor pressure for ascospore discharge (6). Several studies reveal that various genetic and metabolic processes are involved in sexual development and ascospore discharge, such as mating-type (MAT) loci, transcription factors, kinases, certain ion-related transport proteins (such as potassium and calcium ion channels), and lipid metabolism (7–11). In addition, epigenetic mechanisms such as repeat sequence-induced point mutations, meiotic silence of unpaired DNA (MSUD), A-to-I RNA editing, and the perithecia-specific RNA interference (RNAi) have been identified during sexual reproduction of *F. graminearum* (12–16). Interestingly, the perithecia-specific RNAi components *Fgdcl1* and *Fgago2* are involved in the perithecium development and ascospore discharge, but not in asexual development and reproduction (15, 16). Therefore, we are curious about the role of *Fgrdrp*, another important component of RNAi, in the sexual stage-specific RNAi pathway of *F. graminearum*.

RNAi is a conserved eukaryotic gene silencing mechanism mediated at the transcriptional and post-transcriptional levels by small non-coding RNAs of approximately 20 to 30 nucleotides (17, 18). In addition to Dicer and Argonaute, RNA-dependent RNA polymerases (RdRPs) also act as a main player in the RNAi pathway (19, 20). RdRPs contain an RNA-directed RNA polymerase domain and initiate RNAi by producing double-stranded RNA (dsRNA). In *Neurospora crassa*, SAD-1 (an RdRP gene) converts aberrant RNA into dsRNA and interacts with Dicer, Argonaute, and other genes to form a silencing complex in the MSUD pathway; QDE-1, another RdRP, generates dsRNA in the quelling pathway using either single-stranded RNAs (ssRNAs) or single-stranded DNA (ssDNA) as templates (21, 22). In *Mucor circinelloides*, RdRP1 utilizes aberrant transcripts from invasive agents (including plasmas, transposons, and viruses) to generate dsRNA, which is then cleaved by Dcl2 to generate siRNA and transferred to Ago1 (23). Two different small RNAs (sRNAs) have been proposed to participate in RNAi: “primary sRNAs” (derived from the cleavage of the original trigger mediated by Dicer) and “secondary sRNAs” (24). RdRPs are also important for the amplification of RNAi response by increasing the amount of secondary siRNA. For example, RdRP2 of *M. circinelloides* RdRP2 is essential for transgene-induced silencing machinery and the epigenetic RNAi pathway owing to its important role in the generation of secondary sRNAs but minor role in the RdRP-dependent and dicer-independent degradation mechanism (23). In addition, certain RdRPs do not participate or play a minor role in the RNAi pathway, such as *N. crassa* RRP-3 and *M. circinelloides* RdRP3 (25).

In the RNAi pathway, the inducers of sRNA include invasive nucleic acids (such as integrative transgenes, viruses, and transposons) and endogenous transcription (including regular transcription and heterochromatin transcription) (26). In *F. graminearum*, neither *Fgrdrp1* nor *Fgrdrp4* alone plays a key role in the antiviral defense response (27). Non-functional or transiently functional RdRPs of *F. asiaticum* may not be able to maintain exogenous secondary siRNA amplification in the spray-induced gene silencing pathway (28). Nevertheless, the silencing of *F. graminearum* genes by exogenous dsRNA is dependent on DCLs, AGOs, and QIP (29). In *F. graminearum*, *Fgdcl1* and *Fgago2* are involved in ascus development and ascospore discharge by affecting the generation of ex-siRNA and milRNA that regulate ascospore development (15, 16). However, there is little information on the detailed role of *Fgrdrp*s in the biogenesis of perithecium-specific esRNAs. Hence, we generated single-deletion mutants of five *Fgrdrps* (*Fgrdrp1* to *Fgrdrp5*), complementary strains, and double deletion mutant and explored the regulatory mechanisms of *Fgrdrps* by comparing the differences in transcriptome and sRNA production between the deletion mutants and the wild-type strain during sexual development.

## MATERIALS AND METHODS

### Strains and stored conditions

The *F. graminearum* wild-type strain PH-1 (NRRL 31084) (5) and its gene deletion mutants generated in this study were cultured on potato dextrose agar (PDA) plates at 25°C and stored in 20% glycerol solution at −80°C.

### Phylogenetic tree

The nucleic acid and protein sequences of fungal RdRPs were downloaded from NCBI. The conserved domains of protein sequences were analyzed online using Pfam (30) and SMART (31). The alignments of nucleic acid and protein sequences were performed with ClustalW, and phylogenetic tree was constructed with MEGA 7.0 using the neighbor-joining method with 1,000 bootstrap replicates. The interaction network of *F. graminearum* protein was predicted by STRING 11.5 (https://cn.string-db.org/). The minimum required interaction score was 0.7, and the interaction sources included text, experiments, databases, co-expression, and co-occurrence.

### RNA isolation and RT-qPCR analysis

In the asexual stage, RNA samples were isolated at 3 h (mycelial stage) and 24 h (sporulation stage) after incubation in carboxymethyl cellulose (CMC) medium. Seven-day-old carrot agar plate hyphae (0 dpi, days post-perithecium induction) and 7-day-old perithecia (7 dpi) were collected as sexual stage samples. According to the manufacturer’s instructions, total RNA was isolated with the TRIzol reagent (Invitrogen, Carlsbad, CA, USA) and the first-strand cDNA was synthesized by the EasyScript One-step gDNA removal and cDNA Synthesis SuperMix (Transgen-Biotech, Beijing, China). Reverse transcription quantitative PCR (RT-qPCR) was performed on a CFX96 Real-Time PCR Detection System (Bio-Rad, California, USA) with the iTaq Universal SYBR Green Supermix (Bio-Rad, Hercules, CA, USA). The beta-tubulin gene was used as the internal control (32). The relative expression of each gene was calculated with the 2^−ΔΔCt^ method, and the mean and standard deviation were calculated from three biological replicates (33). The relevant primer for RT-qPCR was listed in Table S1.

Quantification of ex-siRNAs was performed as previously reported (15). Small RNAs were enriched from total RNAs using the miRNA isolation kit (OMEGA, Guangzhou, China) according to the manufacturer’s instructions. 100 ng enriched small RNA samples was used for reverse transcription reactions using the miRNA First Strand cDNA Synthesis Kit (Sangon, Shanghai, China). The ex-siRNAs were detected using 25–30 cycles of PCR and separated by 3% agarose gel. The relevant primer for sRNA RT-PCR was listed in Table S1.

### Generation of the deletion mutants and complementation strains

The strategy for gene deletions is based on the split marker system (34). In the single-knockout mutant, the target gene was replaced with the geneticin resistance cassette (gen) or hygromycin resistance cassette (hyg). To generate the double-knockout mutant, two target genes were replaced with hygromycin and geneticin resistance cassette. The hygromycin and geneticin cassettes were amplified from vector pUCH18 and pUCN18 (35, 36). PCR products for targeting deletions were constructed by a slightly modified double-joined PCR. Protoplast preparation and polyethylene glycol-mediated transformation were performed according to the previously published protocol (37). Transformants were transferred to a PDA plate amended with 250 µg/mL hygromycin B (Sigma-Aldrich, St. Louis, MO, USA) or 300 µg/mL geneticin (Sigma-Aldrich, St. Louis, MO, USA) for transformant selection. Transformants were purified by single conidium isolation and stored in 20% glycerol at −80°C. The deletion mutants were identified by PCR and further confirmed by Southern blot. Genomic DNAs of PH-1 and mutants were extracted from mycelium according to the Fusarium laboratory manual and then digested with *HindIII* (Takara, Shiga, Japan) (38). Southern blotting was performed according to the Amersham AlkPhos Direct Labeling and Direction System (GE Healthcare, Little Chalfont, UK). The probe primers are listed in the Table S1.

The *in situ* complementation assays of Δ*Fgrdrp1,* Δ*Fgrdrp2*, and Δ*Fgrdrp5* were performed using the split marker system, as previously described (39). To construct the complementary vector, the entire gene *Fgrdrp1* and *Fgrdrp5* including its promoter region were amplified and cloned into vector pUCH18 using the One Step Cloning Kit (Vazyme-Biotech, Nanjing, China), respectively. And *Fgrdrp2* was cloned into vector pCETNS4 containing geneticin resistance cassette. The complementary fragments were amplified from complementary vectors and gene upstream or downstream fragments by double-joint PCR (40). The fusion constructs were transformed into deletion mutants as described above. The related primers for constructing deletion mutants and complementation strains were listed in Table S1, and the strategy for deletion and complementation was illustrated in Fig. S1.

### Growth rate and conidiation assay

The mycelium growth rate was tested on PDA, minimal medium, and complete medium plates at 25°C (41). Colony morphology was photographed at 4 days. Conidial production was measured in liquid CMC medium (1 g NH_4_NO_3_, 1 g KH_2_PO_3_, 0.5 g MgSO_4_.7H_2_O, 1 g yeast extract, 15 g carboxymethyl cellulose, and 1 L of water), and one mycelial plug was cultured in 20 mL CMC medium at 25°C for 5 days in a shaker (200 rpm). The conidia production was counted by a hemocytometer (42). The lengths of 100 conidia for each strain were randomly measured with Nikon N*i*-U microscopes.

### Plant infection assays

Infection assays on wheat coleoptiles were conducted as previously described (32). Conidia were harvested from 5-day-old CMC cultures and resuspended to 5.0 × 10^5^ conidia/mL in 0.01% Tween 20 solution. Ten wheat coleoptiles of Zhengmai 9023 were inoculated with 2 µL conidial suspension of each strain. Two microliters of 0.01% Tween 20 solution was used as control. The brown lesion length of each strain was measured and photographed at 7 dpi (days post-inoculation).

### Sexual development and ascospore discharge assays

For sexual reproduction, aerial hyphae of 7-day-old carrot agar cultures were pressed down with sterile 2.5% Tween 60 and then incubated under near-UV light (wavelength, 365 nm; Beauty Bright Lighting Electrical Appliance Co. LTD., Zhongshan, China) at 23°C for 7-10 days (38). Perithecia and cirrhi were photographed using a Nikon SMZ25 stereo microscope. To assay the development of ascus, 7-day-old perithecia were gently crushed and observed under Nikon N*i*-U microscopes. Asci were collected for the analysis of various polyols spectrophotometrically according to reference 43. The concentration of glycerol, mannitol, and glucose in asci was measure with the corresponding substance detection kit (mlbio, Shanghai, China).

Ascospore discharges were performed as previously described (44). After incubation for 14 h, the accumulations of ascospores were captured on camera (36). The discharged ascospores were counted as previously described (9). When discharged ascospores were observed on the lids of petri dish, the lid was washed using 1 mL of ddH_2_O to collect the ascospores, and then, the ascospore morphology was observed by a light microscope and counted with a hemocytometer. Addition of exogenous ions to carrot agar cultures for assessing the effect of ions on the level of ascospore discharge, according to reported methods (9, 43) .

### Staining observation

The glycogen staining of asci was performed as previously reported (45). Calcofluor white (CFW) and DAPI staining was performed as previously described (46). To assay the septation of ascospore during ascus development, 7-day-old perithecia were crushed and stained with CFW (20 µg/mL) (Sigma-Aldrich, St. Louis, USA). To assay the nuclear division of ascospore, discharged ascospores were harvested and stained with 4,6-diamidino-2-phenylindole (DAPI, 20 µg/mL) (Sigma-Aldrich, St. Louis, USA). Samples were examined for CFW and DAPI staining signals with a Nikon N*i*-U epifluorescence microscope.

### sRNA-seq and RNA-seq analysis

Perithecia of PH-1, Δ*Fgrdrp1*, Δ*Fgrdrp2*, and Δ*Fgrdrp5* were collected from carrot agar cultures at 7 dpi and 10 dpi, and the total RNA was extracted with TRIzol (Invitrogen, Carlsbad, CA, USA). RNA samples were isolated from two biological replicates for each strain. Small RNAs were extracted from total RNA at 7 dpi using a 15% denaturing polyacrylamide gel. RNA quality was measured using a NanoDrop 2000 spectrophotometer (Thermo Fisher Scientific, Waltham, MA, USA) and Agilent 2100 Bioanalyzer (Agilent, Palo Alto, CA, USA). sRNA and mRNA libraries were constructed as previously described (16). Strand-specific RNA sequencing (RNA-seq) were performed using Illumina NovaSeq 6000 (Personal Bioinformatics Institute, Shanghai, China). Raw data of RNA-seq and small RNA sequencing (sRNA-seq) of *Fgrdrp* mutants have been deposited in NCBI’s Sequence Read Archive (BioProject: PRJNA884510 and PRJNA888203; SRA metadata: PRJNA884510 and PRJNA888203).

With certain modifications, the small RNA analysis was performed as previously reported (15). Clean reads were obtained by removing low-quality reads and adapter sequences using the Fastq toolkit (47). Small sequence reads were completely aligned to the *F. graminearum* PH-1 genome from the Ensembl Fungi database by Bowtie (12, 48). The perfectly matched reads were classified by Bowtie according to *F. graminearum* genomic features such as rRNA, tRNA, exon, intron, untranslated region (UTR), and intergenic region extracted from the .GTF or .GFF file (downloaded from Ensembl Fungi database) using shell script and BEDTools (49). As the information about the UTR region of *F. graminearum* was less confirmed, we took the liberty of expanding the 5′-UTR and 3′-UTR to 500 bp upstream and downstream of gene (started from the start/stop codon), respectively. The reads of ex-siRNAs were normalized according to TPM normalization commonly used for miRNA, where the n_base is 1,000,000 (50, 51). We defined an ex-siRNA as “expressed” in the wild-type strain only when the values of both biological replicates were greater than or equal to 10 TPM and then performed differential expression analysis.

The strand-specific RNA-seq clean reads were mapped to the genome of *F. graminearum* PH-1 by HISAT2, and the mapped counts of each gene were calculated using Stringtie (52, 53). Differential expression analysis of genes was performed with the DEseq2 package (54). Genes with *P*-adj (BH adjusted *P* values) of below 0.05 and |log2FC (fold change)| above 2 were regarded as differentially expressed genes (DEGs). The pathways of DEGs in Kyoto Encyclopedia of Genes and Genomes (KEGG) database were enriched by ClusterProfiler (55, 56), and the *P* value was calculated and subjected to Benjamini-Hochberg correction, with *P* values ≤ 0.05 as a threshold. Heatmap and clustering were performed by ComplexHeatmap (57). Correlation of expression levels between genes and ex-siRNAs was calculated by Hmisc and plotted with Corrplot.

### Statistical analysis

Statistical analyses were performed using Prism 8 (GraphPad Software). Differences between control and multiple treatment groups were detected by Fisher’s LSD. *, **, and *** indicated statistically significant difference at *P* ≤ 0.05, *P* ≤ 0.01, and *P* ≤ 0.001, respectively.

## RESULTS

### Expression pattern and deletion of five *Fgrdrps*

**Fig 1:**
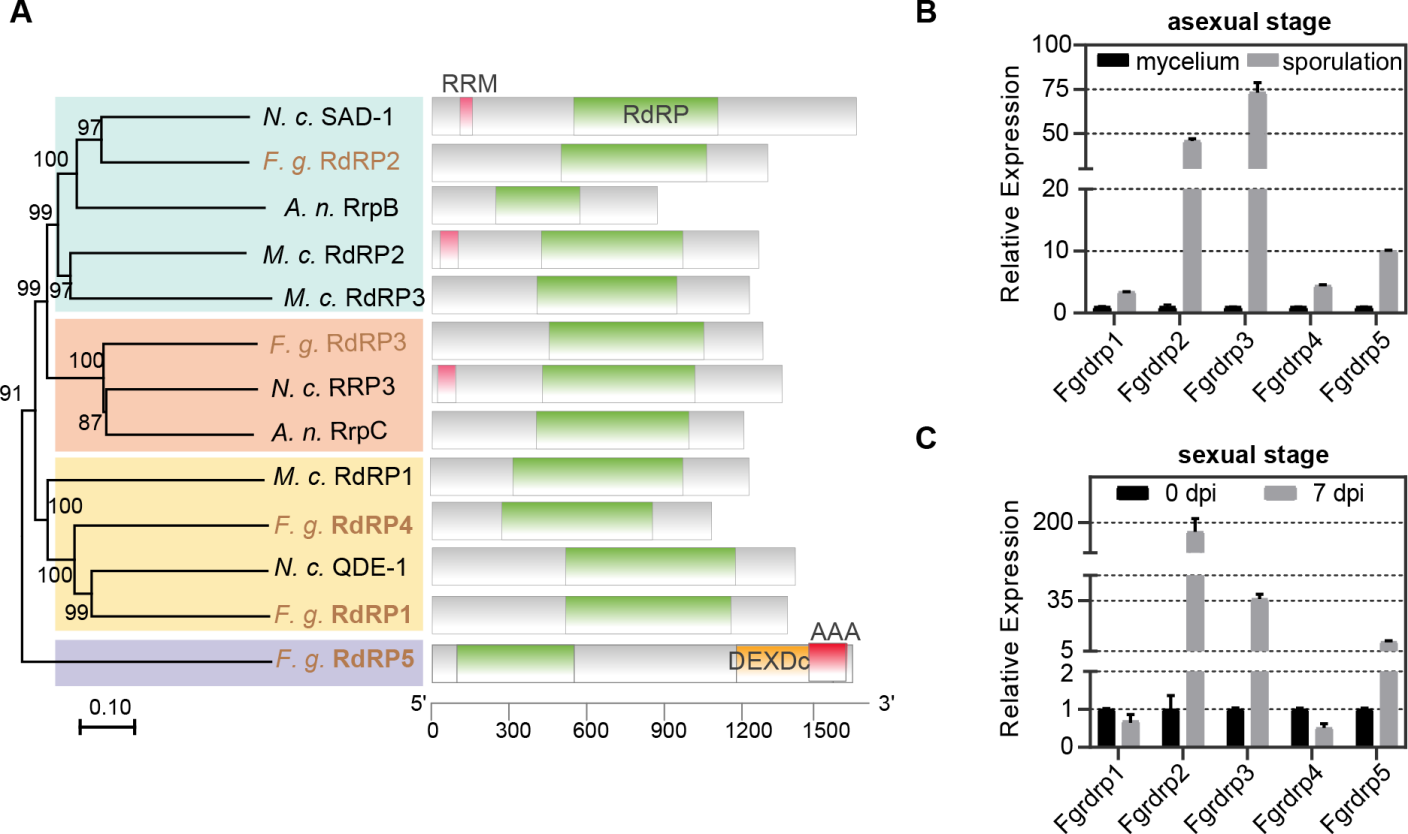
Phylogenetic tree and expression pattern of *F. graminearum FgRdRP*s. (**A**) Phylogenetic relationship and the conserved domains of *F. graminearum* five RdRP proteins and other fungal RdRPs. The alignment was performed with ClustalW, and the MEGA 7.0 was used to perform a 1,000-bootstrap phylogenetic analysis using the neighbor-joining method. *N. c*., *Neurospora crassa*; *A. n*., *Aspergillus nidulans*; *M. u*., *Mucor circinelloides*; SAD-1, suppressor of ascus dominance 1. Domain organization of RdRPs was shown by boxes. RdRP (PFAM accession number: PF05183), RNA-dependent RNA polymerase domain; RRM (SMART accession number: SM00360), RNA recognition motif; DEXDc (SM00487), DEAD-like helicases superfamily; AAA (PF13087), AAA domain; and QDE-1, quelling defective 1. (**B**) Expression levels of *Fgrdrp* genes in the asexual stage. Cultures were collected from YEPD and CMC medium after 24 h inoculation as mycelium (arbitrarily set to 1) and sporulation samples, respectively. (**C**) Expression levels of *Fgrdrp* genes in the sexual stage. Seven days post-perithecium induction, perithecia were collected . The mycelium collected from the carrot plate for 7-day inoculation was considered as the 0-dpi sample (arbitrarily set to 1). The relative mRNA expression of the *Fgrdrp* genes was determined by RT-qPCR. Bars indicate standard deviation from three repeated experiments.

*F. graminearum* contains five RdRPs (FGSG_06504, FGSG_08716, FGSG_01582, FGSG_04619, and FGSG_09076), and the biological functions of four RdRPs (*Fgrdrp1-4*) have been reported in a previous study (29, 58). Phylogenetic tree revealed that *Fgrdrp2* and *Fgrdrp3* were closely related to *N. crassa* SAD-1 and RRP-3, respectively, while *Fgrdrp1*, *Fgrdrp4*, and QDE-1 were clustered in the same clade (Fig. 1A). Interestingly, *Fgrdrp5* was in a separate branch (Fig. 1A). In addition to a conserved RdRP domain, *Fgrdrp5* contains two other unique domains: a DEXDc domain, which is defined as DEAD-like helicases superfamily, extending from positions 1,277 to 1,478 aa (*e*-value: 6.24*e*^−48^), and an AAA domain, which extends from positions 1,475 to 1,677 aa (*e*-value: 2.10*e*^−63^) (Fig. 1A). Compared with the mycelial stage, the expression levels of the five *Fgrdrp*s in the sporulation stage were increased, especially *Fgrdrp2*, *Fgrdrp3*, and *Fgrdrp5*, which were increased by 45.8-, 73.2-, and 10.0-fold, respectively (Fig. 1B). During sexual reproduction, *Fgrdrp2*, *Fgrdrp3*, and *Fgrdrp5* were increased and expressed about 189.7-, 36.1-, and 10.5-fold at 7 dpi, while *Fgrdrp1* (~0.7) and *Fgrdrp4* (~0.5) were decreased (Fig. 1C). Based on these results, we speculated that *Fgrdrp5* may also play an important role in asexual sporulation and sexual reproduction. Therefore, we generated single-deletion mutants in wild-type strain PH-1 and the transformants were screened by PCR and confirmed using southern blots (Fig. S1).

### *Fgrdrp2* plays a minor role in conidial morphology

**Fig 2:**
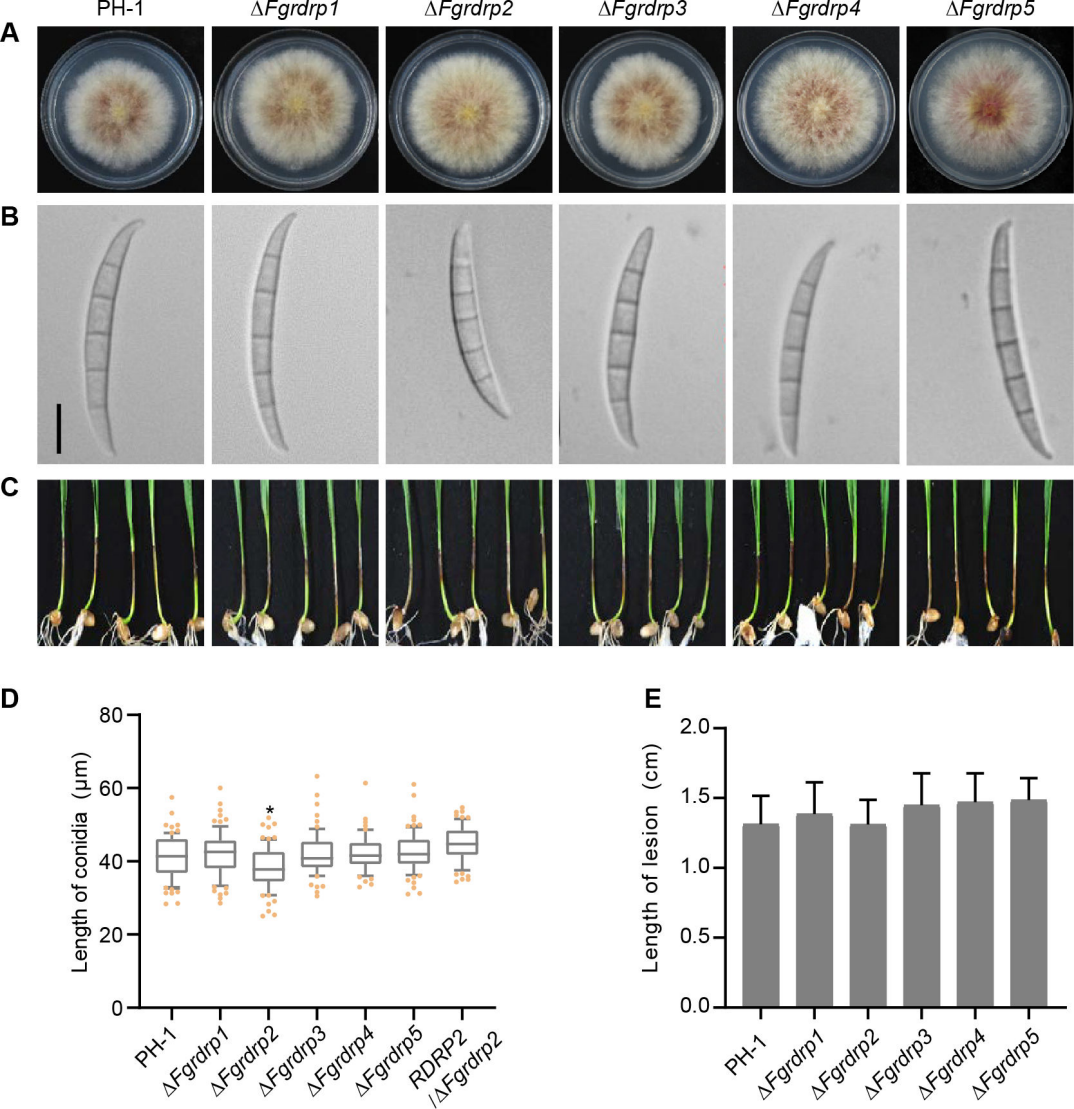
Comparison of colony morphology, conidial morphology, and pathogenicity between the wild-type PH-1 and *Fgrdrp* mutants. (**A**) Four-day-old PDA cultures of the wild-type PH-1 and *Fgrdrp* mutants. (**B**) Conidia morphology of the wild-type PH-1 and *Fgrdrp* mutants. Conidia suspension of each strain was observed after incubation in CMC liquid culture at 25°C for 5 days; bar = 10 µm. (**C**) Wheat coleoptiles infected with PH-1 strain and *Fgrdrp* mutants were observed and photographed. (**D**) Statistics of conidial length. One hundred conidia were measured for each strain. Lines of box-and-whiskers depict 25 to 75 percentile (box), mean, 10 to 90 percentile (whiskers). Points represent the conidial length values outside the 10 to 90 percentile. * indicated significant differences at *P* ≤ 0.05 compared with wild-type PH-1. (**E**) Statistics of the brown lesion length of PH-1 strain and *Fgrdrp* mutants on wheat coleoptiles. Mean and standard deviation were calculated from five biological replicates.

As shown in Fig. 2A and Table S2, there were no apparent differences in colony morphology and growth rate between *Fgrdrp* deletion mutants and the wild-type strain. In 5-day-old CMC cultures, the amounts of conidia produced by these deletion mutants and the wild-type strain were similar (Table S2), whereas the conidial length of Δ*Fgrdrp2* (38.2 ± 5.9) was slightly shorter than that of the wild-type strain (41.5 ± 5.6) (Fig. 2B). When wild-type *Fgrdrp2* allele was re-introduced into Δ*Fgrdrp2*, the conidial length of the complementary transformant *RDRP2/*Δ*Fgrdrp2* was restored to that of the wild- type strain (Fig. 2D). Furthermore, *Fgrdrps* deletion mutants caused typical disease symptoms on wheat coleoptiles and displayed similar lesion lengths to the wild-type strain (Fig. 2C and E). These findings suggested that *Fgrdrp*2 slightly affects conidial morphology and five *Fgrdrp* genes are not essential for vegetative growth and infection.

### *Fgrdrp1, Fgrdrp2*, and *Fgrdrp5* are important for ascospore discharge

**Fig 3:**
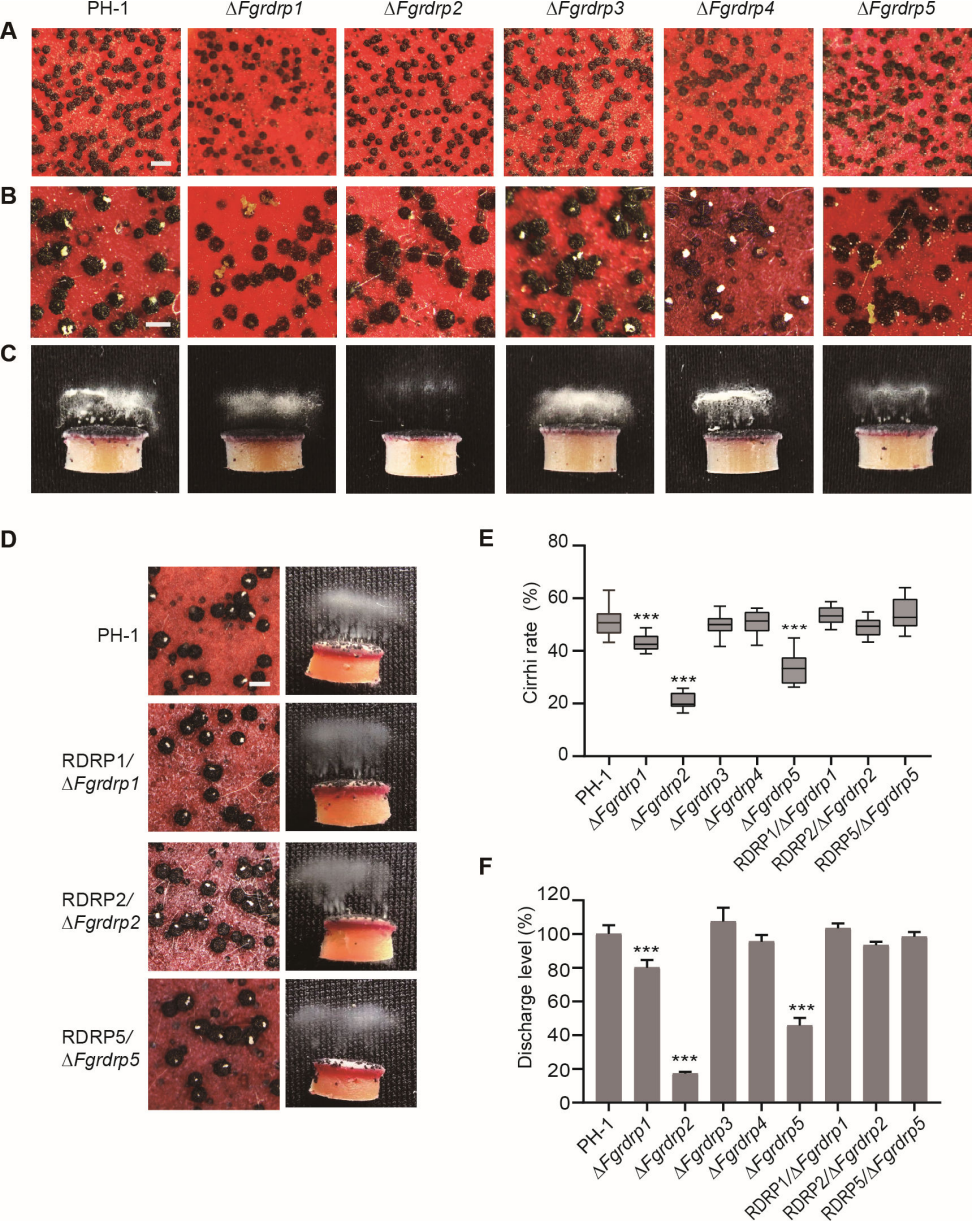
Defects of the *Fgrdrp* deletion mutants in sexual reproduction. (**A**) Perithecia of wild-type PH-1 and *Fgrdrp* mutants were captured at 7 dpi. Bar = 500 µm. (**B**) Perithecia of each strain were examined for cirrhi. The yellow cirrhi was the overflowed ascospores from the ostiole of perithecia at 10 dpi. Bar = 250 µm. (**C**) Ascospore discharge was assayed with perithecia of wild-type PH-1 and *Fgrdrp* mutants collected at 7 dpi. (**D**) Detection of cirrhi and ascospore discharge in wild-type PH-1 and complementary strains. (**E**) Statistics of the cirrhi. The proportion of perithecia with yellow cirrhi was calculated from five representative views under a stereomicroscope. Cirrhi rate (%) = the number of cirrhi/the total number of perithecium × 100%. Lines of box-and-whiskers depict 10 to 90 percentile (box), mean, min to max (whiskers). (**F**) Comparison of discharged ascospores of wild-type PH-1, *Fgrdrp* mutants, and complementary strains. The amount of ascospores discharged of the wild-type PH-1 was used as the 100% discharged level, and the relative discharge levels of the other strains were calculated based on the counts of discharged ascospores. *** indicated significant difference at *P* ≤ 0.001 compared with wild-type PH-1.

To clarify the function of these five *Fgrdrp* genes in the sexual reproduction of *F. graminearum*, the perithecia of these *Fgrdrp*-deletion mutants were induced and examined. On selfing mating plates, normal-sized perithecia were formed by these mutants and the wild-type strain PH-1 (Fig. 3A). At 10 dpi, approximately 50% of perithecia produced cirrhi in the wild-type strain, while the cirrhi rates of Δ*Fgrdrp1*, Δ*Fgrdrp2*, and Δ*Fgrdrp5* were 43%, 21%, and 34%, respectively (Fig. 3B and E). Furthermore, compared with the wild-type strain PH-1 (100%), the capacity of ascospore discharge was notably reduced in Δ*Fgrdrp2* (17.1%) and Δ*Fgrdrp5* (47.6%) and slightly reduced in Δ*Fgrdrp1* (80.1%) (Fig. 3C and F). The levels of cirrhi and ascospore discharge of the complement strains were similar to those of the wild-type strain (Fig. 3D through F). Although *Fgrdrp3* displayed high-level expression in sexual reproduction, there were no significant differences between Δ*Fgrdrp3* and the wild-type strain in cirrhi and ascospore discharge (Fig. 3A through C; Table S2), and the double-deletion mutant Δ*Fgrdrp2/3* produced a similar amount of discharged ascospores as Δ*Fgrdrp2* (Table S2). These results indicated that *Fgrdrp1*, *Fgrdrp2*, and *Fgrdrp5* have a crucial role in ascospore discharge.

### Δ*Fgrdrp1*, Δ*Fgrdrp2*, and Δ*Fgrdrp5* possess low turgor pressure

**Fig 4:**
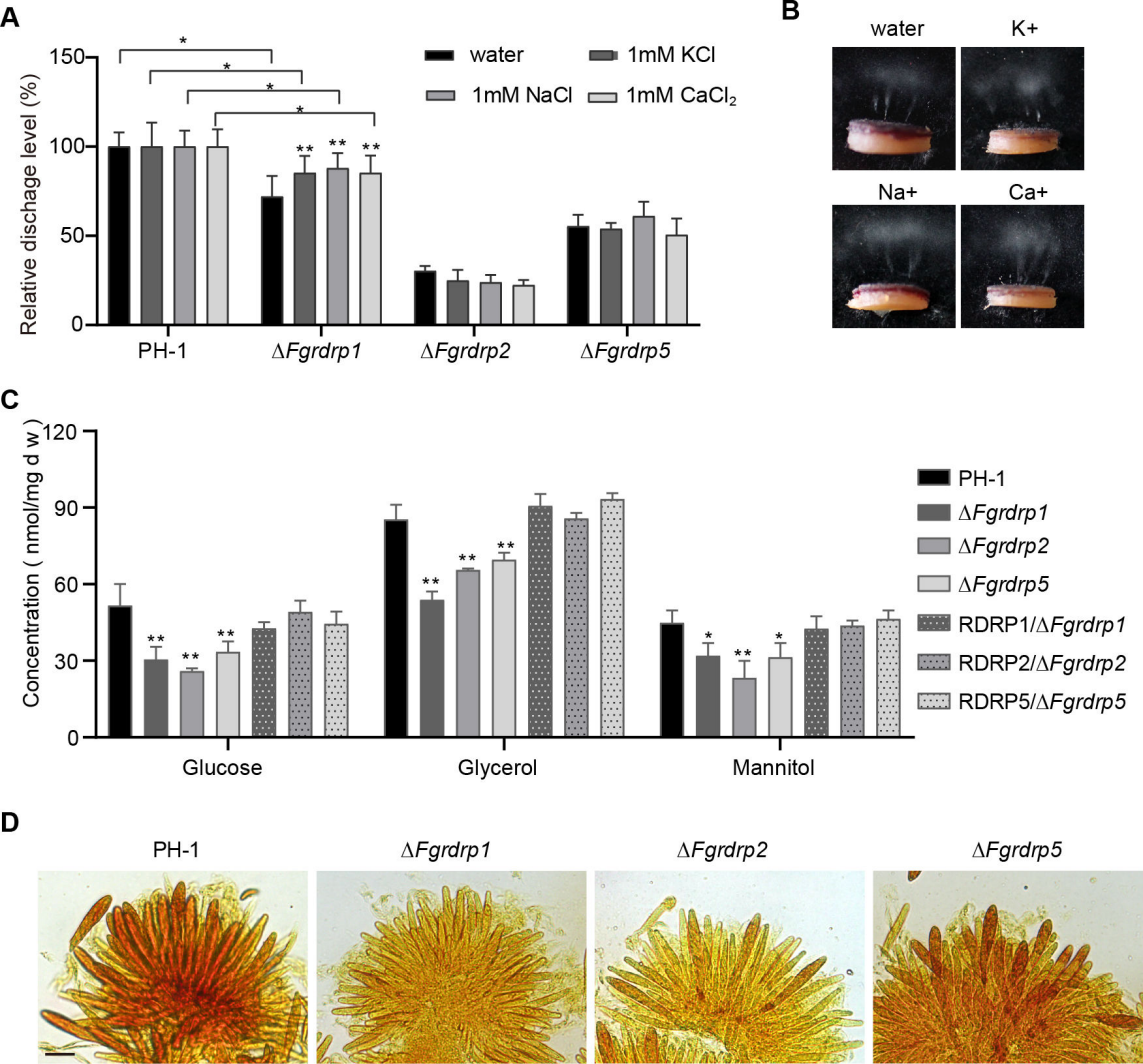
Osmolates in asci were reduced in *Fgrdrp* deletion mutants. (**A**) Comparison of discharged ascospores of the wild-type strain PH-1 and *Fgrdrp* deletion mutants under various ion treatment. The amount of ascospores discharged from the wild-type PH-1 was used as 100% discharged under various ion treatments. (**B**) Ascospore discharge of perithecia harvested from Δ*Fgrdrp1* cultured on carrot agar plates with exogenous ions. (**C**) The concentration of polyols in asci was measured spectrophotometrically. Error bars represent the SD, * indicates significant differences at *P* ≤ 0.05, and ** indicates significant difference at *P* ≤ 0.01. (**D**) Glycogen staining (yellowish-brown) in asci of 7-day-old perithecia. Bar = 20 µm.

In *F. graminearum*, the accumulation of ions in the asci leads to an influx of water, which causes turgor pressure and eventually releases ascospores (6, 59). In order to confirm whether low ion concentrations lead to defective ascospore discharge in Δ*Fgrdrp1*, Δ*Fgrdrp2*, and Δ*Fgrdrp5*, perithecia of these mutants were treated with exogenous ions (K^+^, Na^+^, and Ca^2+^) and their ascospore discharge was assessed. In comparison with the water treatment, the ascospore discharge of Δ*Fgrdrp1* was increased under K^+^, Na^+^, and Ca^2+^ treatments. Nevertheless, under these ion treatments, the discharge capacity of Δ*Fgrdrp1* ascospores was still lower than that of the wild-type strain (Fig. 4A and B). On the other hand, the ascospore discharge defects of Δ*Fgrdrp2* and Δ*Fgrdrp5* were not greatly restored by treatment with exogenous ions (Fig. 4A). These results confirm that low ion concentrations are not responsible for the defective ascospore discharge of Δ*Fgrdrp2* and Δ*Fgrdrp5*, whereas ascospore discharge of Δ*Fgrdrp1* is partially dependent on these ions. Since polyols in asci also contribute to the generation of turgor pressure (43, 59), we detected the concentrations of various polyols (glycerol, mannitol, and glucose) in the asci of Δ*Fgrdrp1*, Δ*Fgrdrp2*, and Δ*Fgrdrp5*. Compared with the wild-type PH-1, the concentrations of polyols were markedly lower in these mutants than the wild-type PH-1 (Fig. 4C). Additionally, staining assay also confirmed reduced glycogen accumulation in asci of Δ*Fgrdrp1*, Δ*Fgrdrp2*, and Δ*Fgrdrp5* (Fig. 4D). Thus, we concluded that *Fgrdrp1*, *Fgrdrp2*, and *Fgrdrp5* contribute to the generation of turgor pressure in a polyol-dependent manner.

### *Fgrdrp1*, *Fgrdrp2*, and *Fgrdrp5* participate in the maturation of ascospores

**Fig 5:**
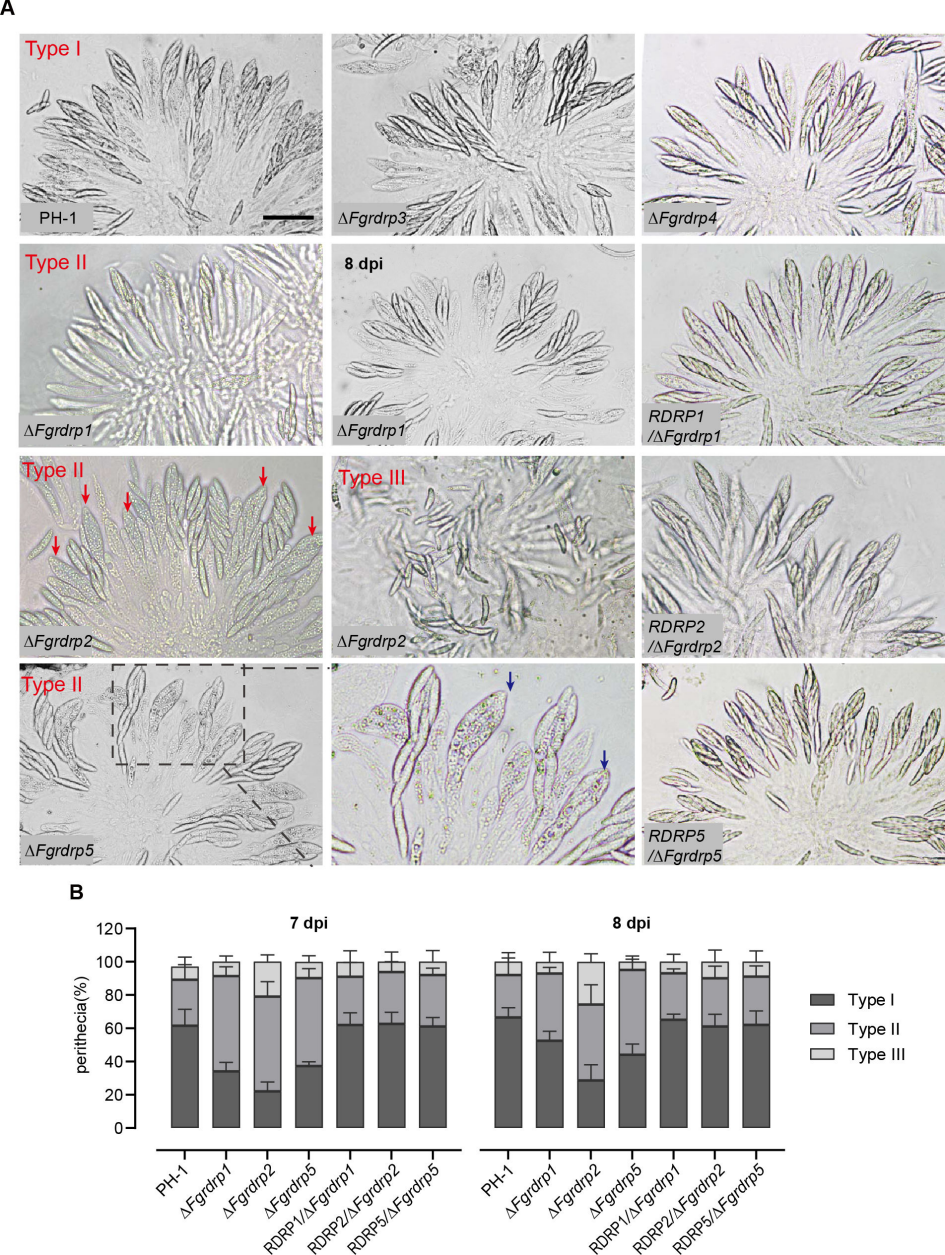
Comparison of the asci and ascospore formation among the PH-1 strain (the wild-type) and *Fgrdrp* mutants. (**A**) The asci rosettes and ascospore were observed at 7 dpi. The red arrows indicate the asci containing immature ascospores in Δ*Fgrdrp2*. Asci containing abnormal ascospores in Δ*Fgrdrp5* are indicated by blue arrows. Bar = 25 µm. (**B**) Percentage of three types of asci rosettes. Types I, II, and III indicate mature, immature, and scattered asci rosettes, respectively. One hundred perithecia were assessed for each strain with three biological replicates.

**Fig 6:**
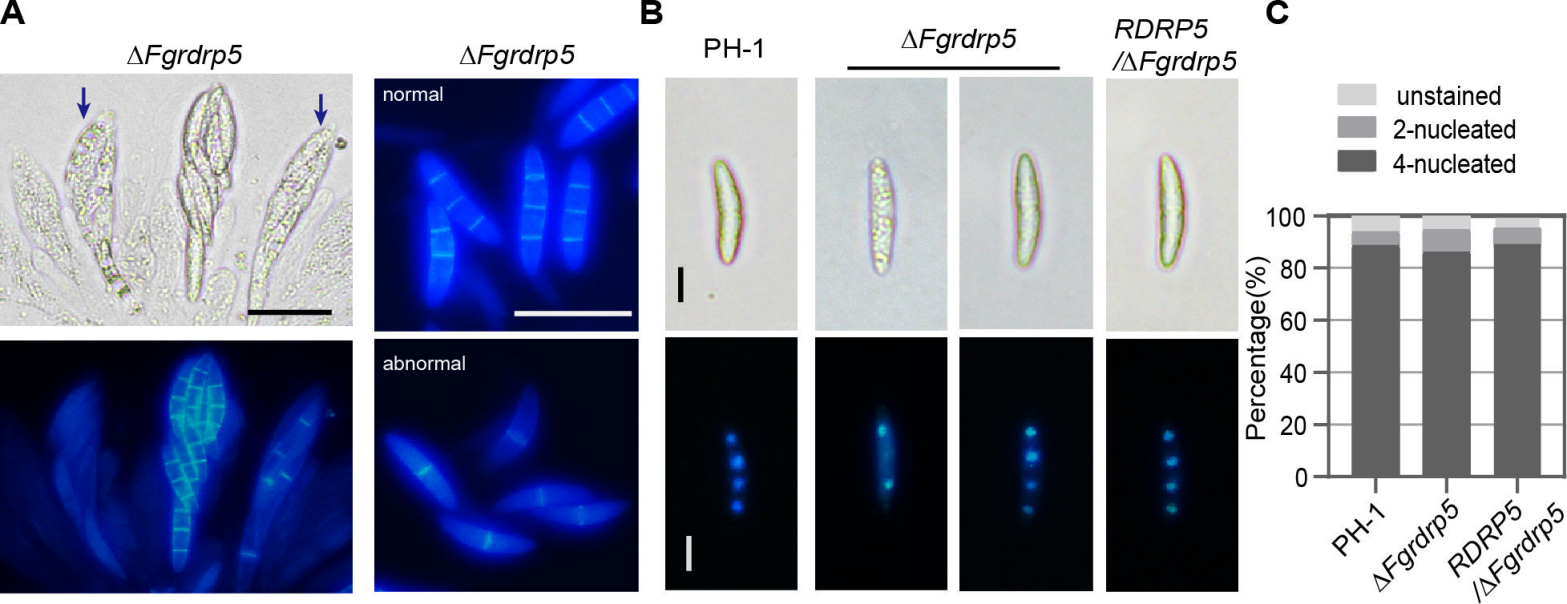
Ascospores of Δ*Fgrdrp5* mutant were stained with CFW (**A**) or DAPI (**B**) and examined under light microscopy and epifluorescence microscopy. (**A**) CFW staining of asci and scattered ascospores. The asci was dissected from 7-day-old perithecia. Blue arrows indicate the asci containing abnormal ascospores. Bar = 20 µm. (**B**) DAPI staining of ejected ascospores. Bar = 5 µm. (**C**) Percentage of the ejected ascospores. The ejected ascospores were stained by DAPI and categorized under epifluorescence microscope.

Since osmolytes associated with turgor pressure usually accumulate after the maturation of ascospores (6, 59), we dissected 7-day-old perithecia to observe and evaluate the development of asci and ascospores. The asci of wild-type strain PH-1, Δ*Fgrdrp3*, and Δ*Fgrdrp4* was dominated by mature asci (Type I) containing eight visible spindle-shaped ascospores (Fig. 5A and B). Fewer spindle-shaped ascospores were observed in the asci of Δ*Fgrdrp1* at 7 dpi, whereas the proportion of mature asci was increased at 8 dpi (Fig. 5A and B). In Δ*Fgrdrp2*, approximately 21% of the perithecia contained scattered ascospores (Type III) and 57% contained loosely arranged and immature ascospores (Fig. 5A and B). Abnormal ascospores with a rough surface resembling vacuolization were observed in approximately 53% perithecia of Δ*Fgrdrp5* (Fig. 5A and B). When stained with CFW, one septum per ascospore (two celled) was observed in abnormal asci, while three septa per ascospore (four celled) were observed in normal asci (Fig. 6A). In addition, in both abnormal and normal ascospore, each cell had one nucleus (Fig. 6B). Further counting of discharged ascospores revealed that the majority (86.1%) of Δ*Fgrdrp5* ascospores were four celled four nucleated, similar to those of wild-type PH-1 (88.6%) (Fig. 6C), implying that the discharged ascospores are mainly derived from mature ascospores and these two-celled ascospores may be immature ascospores, which delay the accumulation of osmolytes in Δ*Fgrdrp5* asci, causing a decrease in discharge. Taken together, these results indicate that *Fgrdrp1*, *Fgrdrp2*, and *Fgrdrp5* participate in the maturation of ascospores.

### *Fgrdrp1*, *Fgrdrp2*, and *Fgrdrp5* play roles in the accumulation and biogenesis of dicer-dependent ex-siRNAs

**Fig 7:**
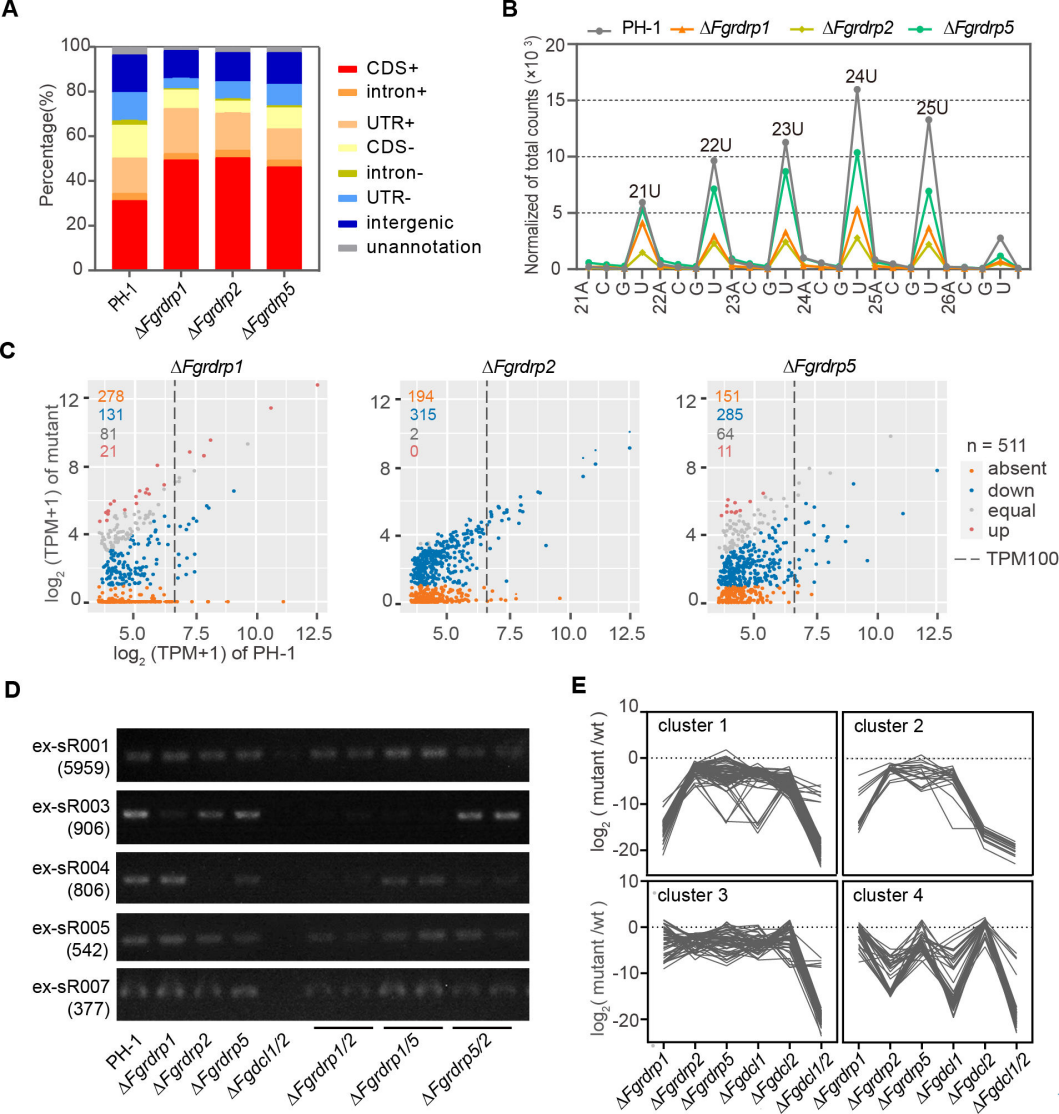
Characterization of small RNAs in the wild-type strain PH-1 and *Fgrdrp* deletion mutants. (**A**) The percentages of sRNA reads mapped to various genomic regions of *F. graminearum*. Exons include the protein coding region (CDS) and the UTR portion. (**B**) Length distribution and nucleotide preference of the 5′ end of ex-siRNAs in the wild-type strain PH-1 and *Fgrdrp*-deletion mutants. The total reads of ex-siRNAs were normalized by TPM (transcripts per million). (**C**) Expression levels of ex-siRNAs (TPM > 10) in wild-type strain PH-1 (abscissa) and *Fgrdrp* deletion mutants (ordinate) at 7 dpi. The ex-siRNAs with a fold change less than 2 were treated as equally expressed sRNAs. The colored numbers indicate the amount of different types of ex-siRNAs. (**D**) Detection of ex-siRNAs in *Fgrdrp* single- and double-deletion mutants. 100 ng enriched sRNA sample was used in the reverse transcription reaction for each strain; numbers in parentheses indicate the TPM value of ex-siRNA; the ex-siRNAs were quantified using stem-loop RT-PCR and detection by 3% agarose gel. (**E**) Expressed clusters of ex-siRNAs in *Fgdcl-* and *Fgrdrp-*deletion mutants. ex-siRNAs were clustered based on the k-means clustering algorithm. The TPM values of the absent ex-siRNA were replaced with 1*10^−4^.

To examine whether *Fgrdrp*s process the perithecium-specific sRNAs, we isolated sRNA sample from 7-day-old perithecia of single-deletion mutants (Δ*Fgrdrp1*, Δ*Fgrdrp2*, and Δ*Fgrdrp5*) and the wild-type strain and performed sRNA-seq analysis. Total reads were mapped to the *F. graminearum* genome, and then, structural non-coding RNAs (rRNA and tRNA) were removed (Table S3). In comparison with the wild-type strain (27.3%), the proportions of sRNAs mapped to the antisense strand of exon were obviously decreased in Δ*Fgrdrp1* (13.79%), Δ*Fgrdrp2* (13.28%), and Δ*Fgrdrp5* (18.91%) (Fig. 7A; Table S4). As noted in a previous report, these sRNAs were reported as exonic siRNAs (ex-siRNAs) and were enriched in 24-nt reads with 5′-U (15). After the normalization of total reads, a decreasing trend in 5′-U reads of 21–25 nt was observed in these *Fgrdrp* deletion mutants, with the most pronounced decrease in Δ*Fgrdrp2* (decreased by about 80%), followed by Δ*Fgrdrp1* (~70%) and Δ*Fgrdrp5* (~32%) (Fig. 7B). To further characterize the expression level of each ex-siRNA (the ex-siRNAs reads with TPM > 10 in the wild-type strain) in *Fgrdrp*-deletion mutants, we categorized the ex-siRNAs and plotted scatterplots. The ex-siRNAs with TPM values less than 1 in the mutant and greater than 10 in the wild-type strain were treated as absent ex-siRNAs. Although more ex-siRNAs were absent in Δ*Fgrdrp1*, the majority of these absent ex-siRNAs were lowly expressed (TPM < 100) in the wild-type strain, and only a few of absent ex-siRNAs shared with other *Fgrdrp* mutants (Fig. 7C; Fig. S2A and B). The ex-siRNAs with a fold change more than two were treated as up- or downregulated ex-siRNAs. As shown in Fig. 7C, equally expressed and upregulated ex-siRNAs were present in Δ*Fgrdrp1* (102/511) and Δ*Fgrdrp5* (75/511), whereas almost all ex-siRNAs were markedly downregulated or absent in Δ*Fgrdrp2* (509/511), implying that the deletion of *Fgrdrp2* interferes with the accumulation of most ex-siRNAs. Since a portion of ex-siRNAs was still expressed in these single-deletion mutants, especially the highly expressed ex-siRNAs (TPM ≥ 100), we generated the double-deletion mutants (Δ*Fgrdrp1/2*, Δ*Fgrdrp5/2*, and Δ*Fgrdrp1/5*) to investigate whether the biogenesis of these ex-siRNAs depends on two *Fgrdrps*. The abundance of ex-siRNAs in single-deletion mutant determined by stem-loop RT-PCR was similar to the results of sRNA-seq (Fig. 7D). The accumulations of ex-siRNAs (ex-sR001, ex-sR005, and ex-sR007), which were present in all three single-deletion mutants, were reduced but not absent in *Fgrdrp* double-deletion mutants (Fig. 7D), meaning that the biogenesis of these ex-siRNAs require more than two *Fgrdrp*s.

Similar to the biogenesis pathway of most esRNAs, the biogenesis of ex-siRNAs in *F. graminearum* depends on *dicer* gene (15). To investigate whether *Fgrdrp*s affect these *dicer*-dependent ex-siRNAs, we downloaded previously published sRNA-seq data of *Fgdcl* mutants from Sequence Read Archive (BioProject: PRJNA431527) and re-analyzed (16). The clustering results showed that ex-siRNAs absent in Δ*Fgrdrp1* and Δ*Fgrdrp2* were also absent in Δ*Fgdcl2* and Δ*Fgdcl1* (cluster 2 and cluster 4), respectively, but the majority of ex-siRNAs absent in Δ*Fgrdrp1* were only lost in Δ*Fgdcl1/2* (cluster 1) (Fig. 7E). In addition, the ex-siRNAs expressed in all three *Fgrdrp* single-deletion mutants were also absent only in Δ*Fgdcl1/2* (cluster 3) (Fig. 7E). Overall, almost all ex-siRNAs were extremely reduced or absent in Δ*Fgdcl1/2* (Fig. 7E). Correlation analysis of sRNA expression changes further confirmed the positive relationships between *Fgrdrp2* and *Fgdcl1* and *Fgrdrp1* and *Fgdcl2* (Fig. S2C). The results of stem-loop RT-PCR also demonstrated that *Fgdicer*s are required for the biogenesis of selected ex-siRNAs, as the selected ex-siRNAs were absent in Δ*Fgdcl1/2* (Fig. 7D). In conclusion, *Fgrdrp1*, *Fgrdrp2*, and *Fgrdrp5* are involved in the biogenesis and accumulation of *dicer*-dependent ex-siRNAs.

### *Fgrdrp*s negatively regulate the ex-siRNAs corresponding genes

**Fig 8:**
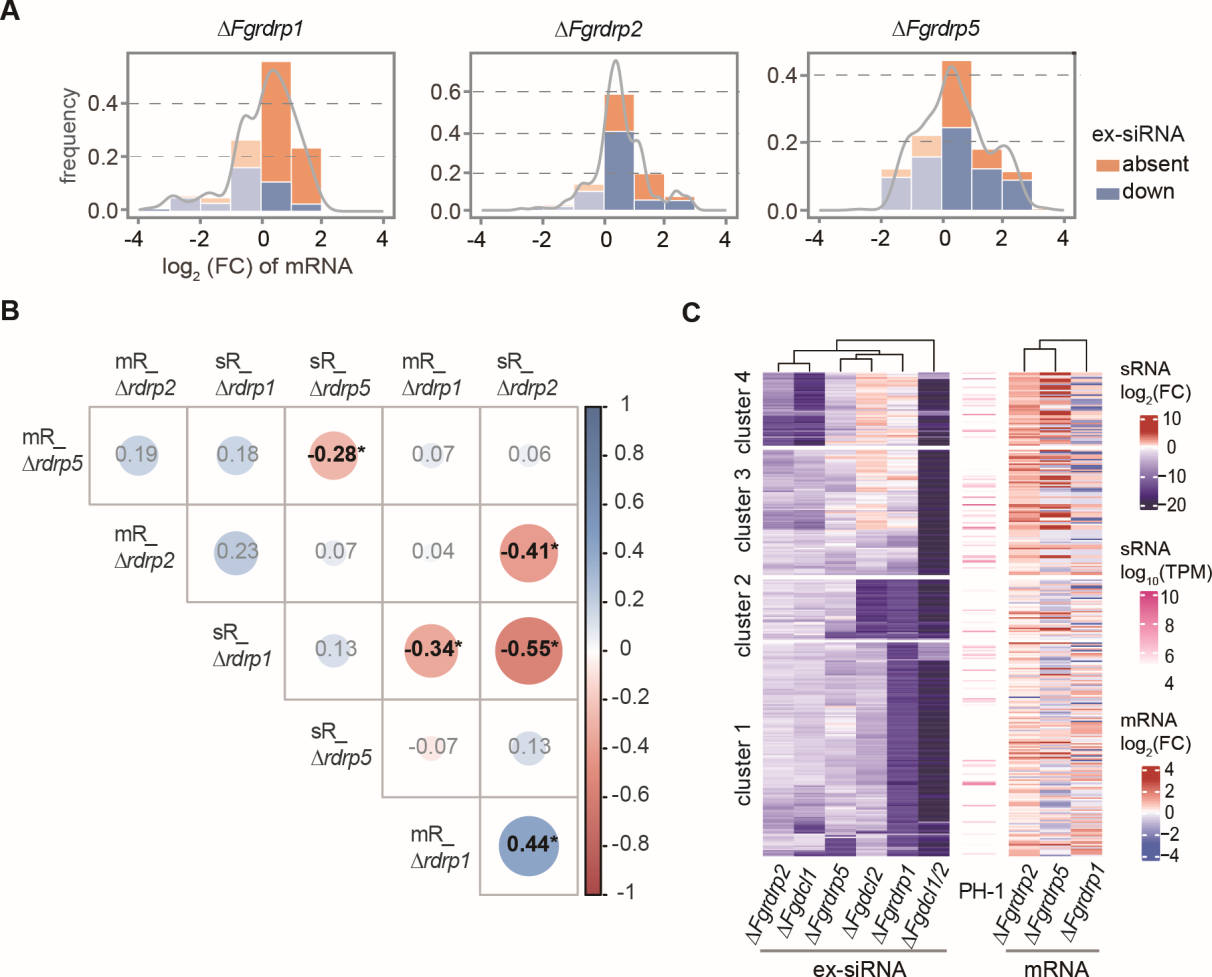
Correlation of expression changes between mRNAs and ex-siRNAs. (**A**) Transcript levels of mRNAs corresponding to absent and downregulated ex-siRNAs. The lower-transparency colors indicate the log2(FC) values less than 0. The gray lines present the density trend of transcript levels corresponding to combined downregulated and absent ex-siRNAs. (**B**) Correlation analyses of expression changes between mRNAs and ex-siRNAs. Pearson correlation coefficients were calculated based on the log2 value of the fold change between mutants and wild-type strain. The TPM values of ex-siRNA derived from the same corresponding gene were combined for the calculation. The light-colored numbers indicate coefficient values with *P* values less than 0.05. * indicates *P* ≤ 0.05. (**C**) Expression heatmaps of sRNA and mRNA. The clustering distance method was “Euclidean,” and ex-siRNAs were clustered by k-means (*n* = 4).

In previous studies, the ex-siRNAs negatively regulate the transcription levels of the corresponding genes and some of them are involved in sexual development (15). To investigate the transcript levels of genes corresponding to downregulated and absent ex-siRNAs in *Fgrdrp*s deletion mutants, we performed RNA-seq with RNA samples isolated from 7-day-old perithecia (Table S5). In these deletion mutants, especially in Δ*Fgrdrp1* and Δ*Fgrdrp2*, the genes corresponding to the absent and downregulated ex-siRNAs were predominantly upregulated, in spite of the fact that most of them showed a smaller fold change (log2 fold change less than 2) (Fig. 8A). The correlation coefficient further confirmed that ex-siRNAs negatively regulated the expression level of corresponding genes, and the coefficient values of Δ*Fgrdrp1*, Δ*Fgrdrp2*, and Δ*Fgrdrp5* were –0.34, –0.41, and −0.28, respectively (Fig. 8B). As shown in Fig. 8C, the significantly upregulated genes of Δ*Fgrdrp2* and Δ*Fgrdrp5* were mainly concentrated in the corresponding genes of cluster 3 and cluster 4 ex-siRNAs, while the upregulated corresponding genes of Δ*Fgrdrp1* were distributed in cluster 1. Among these upregulated ex-siRNA corresponding genes, FGSG_02767 and FGSG_06565 were involved in signal transduction pathways (Fig. S2D). FGSG_05418 and FGSG_02800, also labeled as ex-siRNA corresponding genes and associated with ascospore formation, were upregulated in these three *Fgrdrp* mutants and Δ*Fgrdrp2*, respectively (Fig. S2D) (11, 15, 60). The transcript levels of ex-siRNA corresponding genes were further verified by RT-qPCR (Fig. S2E).

### *Fgrdrp*s regulate the expression of genes involved in sexual development

**Fig 9:**
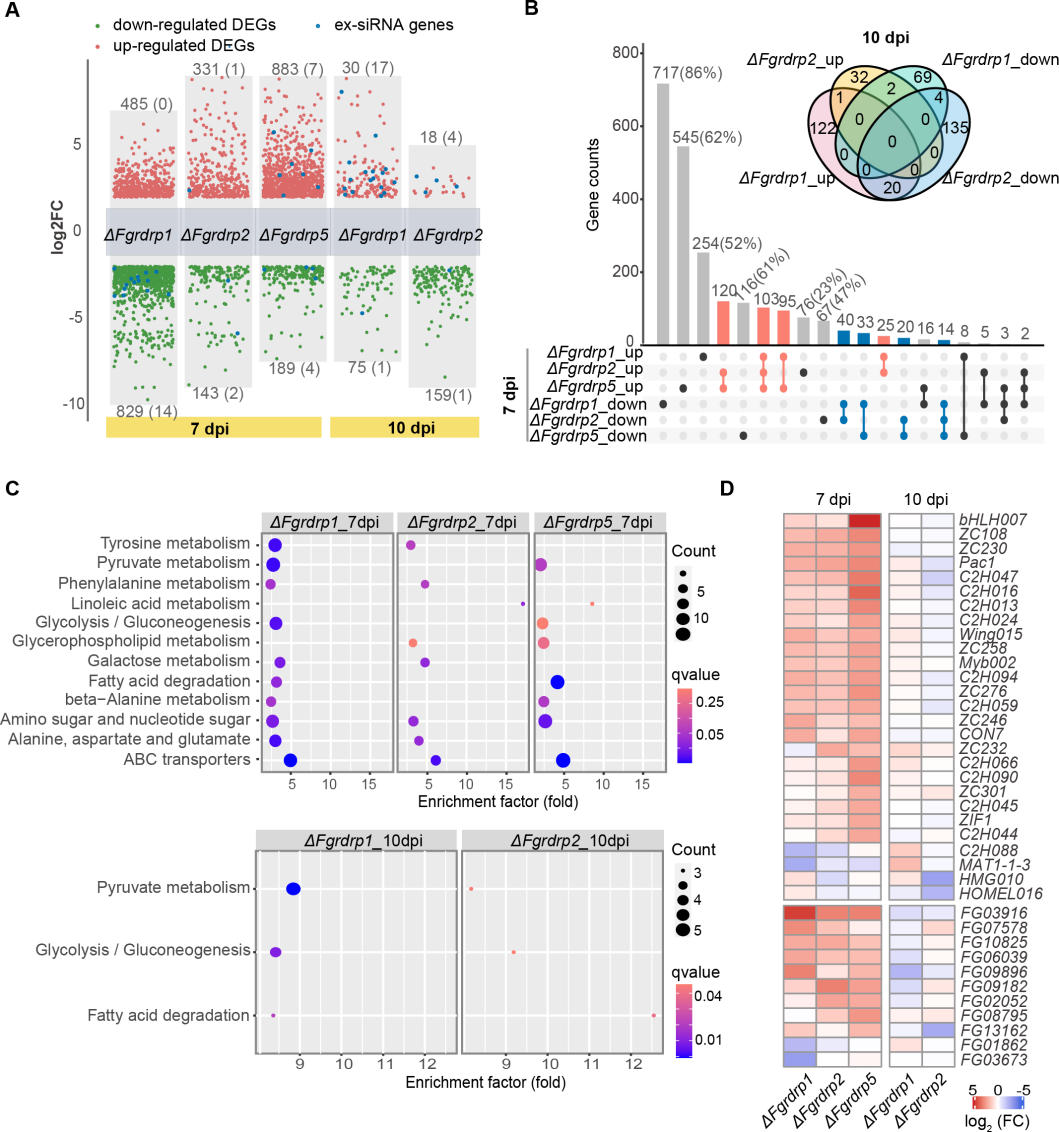
Characterization of DEGs in *Fgrdrp*-deletion mutants. (**A**) Volcanic plot of DEGs in *Fgrdrp*-deletion mutants at 7 dpi and 10 dpi. Numbers in parentheses indicated the counts of ex-siRNA corresponding genes. (**B**) Upset and Venn diagram of DEGs in *Fgrdrp* mutants at 7 dpi and 10 dpi. The intersections of the up- and downregulated DEGs were marked in pink and blue, respectively. (**C**) Common KEGG pathways enriched by DEGs. KEGG pathways with *P* values ≤ 0.05 are shown. (**D**) The expression heatmap of DEGs annotated as transcription factors or sex-related genes.

To explore the effect of *Fgrdrp* deletion on other functional genes, we considered genes with false discovery rate (FDR) less than 0.05 and the absolute value of the log2 of fold change greater than 2 as DEGs. In these 7-dpi samples, the downregulated DEGs in Δ*Fgrdrp1* were more than upregulated, while the proportions of upregulated DEGs in Δ*Fgrdrp2* and Δ*Fgrdrp5* were approximately 79% and 60%, respectively (Fig. 9A). Since Δ*Fgrdrp1* and Δ*Fgrdrp2* delayed ascospore maturation, we also collected the RNA samples from 10-day-old perithecia for differential gene expression analysis. In Δ*Fgrdrp1* and Δ*Fgrdrp2*, the amount of DEGs (mutant/wild type) at 10 dpi was significantly lower than that at 7 dpi, suggesting that these mutants are slightly different from the wild-type strain at 10 dpi, while the expression changes of DEGs at 7 dpi may be close to the defective asci or ascospore discharge (Fig. 9A). In addition, in Δ*Fgrdrp1* and Δ*Fgrdrp2*, more DEGs in the 10-dpi samples were labeled as ex-siRNA-associated genes compared with 7-dpi samples, implying that ex-siRNAs may be accumulated and exert regulatory roles in mature ascospore (Fig. 9A). Upset plot reflected that a multitude of DEGs in Δ*Fgrdrp1* and Δ*Fgrdrp5* did not intersect with other mutants, whereas only 23% upregulated and 47% downregulated DEGs were unique to Δ*Fgrdrp2* (Fig. 9B). In Δ*Fgrdrp2*, nearly 67% (223/331) upregulated DEGs were shared with Δ*Fgrdrp5*, and these common upregulated DEGs were significantly enriched in membrane transport (ABC transporters), lipid metabolism (linoleic acid metabolism), and glycosyltransferases (Fig. 9B; Fig. S3). At 7 dpi, certain KEGG pathways were co-enriched in two or three mutants; for instance, galactose metabolism was enriched in Δ*Fgrdrp1* and Δ*Fgrdrp2*, pyruvate metabolism, glycolysis/gluconeogenesis, and fatty acid degradation were enriched in Δ*Fgrdrp1* and Δ*Fgrdrp5*, and ABC transporter was enriched in all three mutants (Fig. 9C). These results signified that *Fgrdrps* regulate the metabolism pathways and transport of lipid- and sugar-related substances, which account for the reduced accumulation of osmolytes in the asci of these mutants. Surprisingly, the deletion of *Fgrdrp5* may affect pre-rRNA processing, as ribosome biogenesis is markedly enriched in Δ*Fgrdrp5* (Fig. S3). Furthermore, 90 DEGs were annotated as transcription factors (TFs), and 27 of them were associated with sexual development. Although most of the TFs were significantly induced, certain TFs affecting perithecia maturation or ascospore discharge were downregulated in *Fgrdrp* mutants, for example, *MAT-1-1-3* in Δ*Fgrdrp1* and Δ*Fgrdrp5*, *HMG010* (FGSG_01366) and *HOMEL016* (FGSG_06966) in Δ*Fgrdrp2*, and *C2H088* (FGSG_10470) in Δ*Fgrdrp1* and Δ*Fgrdrp2* (Fig. 9D; Table S6) (61). In addition, two downregulated DEGs (FGSG_01862 and FGSG_03673) of Δ*Fgrdrp1* were closely related to sexual development in *F. graminearum* (Fig. 9D; Table S6). In particular, the deletion of FGSG_01862 delayed perithecia maturation (10). Hence, reduced expression of these genes may be related to the defects of Δ*Fgrdrp1*, Δ*Fgrdrp2*, and Δ*Fgrdrp5* in perithecia maturation or ascospore discharge.

## DISCUSSION

In contrast to other RNAi components, RdRP proteins are not as widely distributed and evolutionary conserved in eukaryotes, such as *Aspergillus nidulans* lost the putative orthologs of *N. crassa* RdRP QDE-1 and *A. fumigatus* lacks an RRP3-like RDRP (62, 63). In this work, *Fgrdrp2* and *Fgrdrp3* were homologous to *N. crassa* SAD-1 and RRP-3, respectively, and *Fgrdrp1*, *Fgrdrp4*, and QDE-1 were evolutionarily similar (Fig. 1A), suggesting *F. graminearum* RdRPs are relatively intact during evolution. In filamentous fungus, *N. crassa*, quelling and MSUD are two typical RNA silencing processes and require the involvement of QDE-1 and SAD-1, respectively (64–66). The MSUD mechanism is confirmed in *F. graminearum*, and *Fgsad-1* (*Fgrdrp2*) is involved in this process (13). However, MSUD is uncommon during normal sexual reproduction because of the homothallic mating type of *F. graminearum*, which produces less invasive DNA. According to the results of the expression pattern, we speculated that *Fgrdrp2*, *Fgrdrp3*, and *Fgrdrp5* may play roles in conidiation and sexual reproduction. However, the further biological investigations revealed that *Fgrdrp2* regulates conidial length as well as maturation and release of ascospores, whereas *Fgrdrp5* only acts on the maturation and release of ascospores (Fig. 2 and 3). It is surprising that *Fgrdrp3* did not play an important role in sporulation and sexual reproduction, at least under the conditions we tested. In previous studies, *Fgdcl* and *Fgago*, which were significantly expressed in conidia compared with those in mycelia, did not significantly affect conidial morphology and production, but *Fgdcl2* and *Fgago1* were essential for the hpRNA-induced gene silencing in the asexual stage of *F. graminearum* (58). Therefore, we hypothesize that these highly expressed *Fgrdrp* genes may play roles in other conditions that we did not test, such as the hpRNA-induced gene silencing pathway or other environments; thus, further studies are required.

In most esRNA biogenesis pathways, dsRNA precursors are cleaved by RNase III Dicer enzymes to generate small RNA duplexes and then loaded onto RNA-induced silencing complex (RISC) with Argonaute protein as the core catalytic component (19, 20). RdRPs, which convert ssRNA into dsRNA and amplify the sRNA signal, also act as a main player (19). However, the nature and number of the proteins involved in the RNAi pathway vary with sRNA types. For example, four classes of *M. circinelloides* exonic siRNAs have been reported based on the proteins involved in their biogenesis: Dcl2 and RdRP2-dependent class I, Dcl2 and RdRP1-dependent class II, Dcl1, Dcl2 and RdRP1 or RdRP2-dependent class III, Dcl1, and RdRP1 or RdRP2-dependent Class IV (67). Previous studies have confirmed the presence of ex-siRNA and milRNA in *F. graminearum*, and *Fgdcl2* is involved in the generation of milRNAs in the asexual stage, while *Fgago2* and *Fgdcl1* cooperate with *Fgdcl2* and play a crucial role in the biogenesis of sex-specific esRNAs (15, 16, 58). In Δ*Fgrdrp1*, Δ*Fgrdrp2*, and Δ*Fgrdrp5*, although most of the *Fgdcl1/2*-dependent ex-siRNAs were not absent, their accumulations were significantly reduced compared with the wild-type strain (Fig. 7). In addition, certain ex-siRNAs, which were present in single-deletion mutants of these three *Fgrdrp*s, are not lost in *Fgrdrp*s double-deletion mutants (Fig. 7D). Hence, the biogenesis of *Fgdcl1/2*-dependent ex-siRNA requires more than two *Fgrdrp*s, and all three *Fgrdrp*s are involved in the accumulations of ex-siRNA. Co-analysis of sRNA data between *Fgrdrp*s and *Fgdcl*s deletion mutants revealed a positive correlation between Δ*Fgrdrp1* and Δ*Fgdcl2* and Δ*Fgrdrp2* and Δ*Fgdcl1* (Fig. 7E and S2C). In previous studies, *Fgdcl1*, a component partially affecting ascospore maturation and discharge, was partially involved in the biogenesis of ex-siRNAs, whereas in Δ*Fgdcl1/2*, ex-siRNAs were extremely lacking and ascospore development and discharge were completely inhibited (15, 16). In our study, among these five *Fgrdrp*s, *Fgrdrp*2 showed the most pronounced effect on ascospore discharge, followed by *Fgrdrp*5 and *Fgrdrp*1 (Fig. 3). We speculate that the defective sexual development of *Fgrdrp*-deletion mutants caused a partial reduction in ex-siRNAs and led to a correlation between *Fgrdrp* and *Fgdcl* in ex-siRNAs. In fungi, certain RNA helicases also play an important role in RNA silencing. For example, the putative RNA helicase SAD-3 mediates MSUD in *N. crassa*; *Hrr1*, an RNA helicase in *Schizosaccharomyces pombe*, is required for RNAi-mediated heterochromatin assembly; *rnhA*, containing a DEAD-like helicase superfamily domain, is essential for epimutation pathway but not for exogenously activated RNAi (25, 60, 68). In *C. elegans*, RDE-12, which contains a DEAD-box helicase domain, promotes secondary siRNA synthesis by coordinating RDE-10 and RRF-1 (RdRP) (69). Therefore, we will further explore whether the DEAD domain of *Fgrdrp5* also plays a role in the accumulation of ex-siRNAs or other mechanisms.

Previous studies have shown that conidial production is reduced in all *Fgrdrp*s mutants under low light (29). In our study, the conidial length of Δ*Fgrdrp2* was shorter than the wild type in the dark (Fig. 2B). It suggests that the initiation of the *rdrp2*-dependent RNAi pathway may differ in response to light changes during sporulation. Compared with the asexual development stage, *Fgrdrp1*, *Fgrdrp2*, and *Fgrdrp5* played more important roles in sexual reproduction. The discharged ascospores of Δ*Fgrdrp1*, Δ*Fgrdrp2*, and Δ*Fgrdrp5* were all decreased (Fig. 3C). In Δ*Fgrdrp1*, fewer ascospores were ejected possibly due to delayed ascospore development as well as defects in lipid metabolism, carbohydrate metabolism (glycolysis/gluconeogenesis), and ABC transporters. In *F. graminearum*, carbohydrate and lipid metabolism, particularly fatty acids, sucrose, glucose, and mannitol, are critical for perithecia development and ascospore discharge, as these substances serve as energy sources for asci development and facilitate the generation of turgor pressure required for ascospore discharge (8, 45, 59). ABC transporters are ubiquitous proteins that facilitate the transmembrane transport of various substances, and certain genes function in the sexual development of fungi such as *F. graminearum Arb1*, *Colletotrichum gloeosporioides CgABCF2*, and *Podospora anserina pABC1* and *pABC2* (70–72).

Among these *Fgrdrp*s deletion mutants, Δ*Fgrdrp2* was the mutant with the most pronounced reduction in ascospore discharge (Fig. 3E), one of the reasons being immature or scattered ascospores (Fig. 5A and B). Similarly, *AMD-1* and *GEA-1* deletion mutants showed a decreased ability to discharge ascospores due to defects in ascus wall development (73, 74). Therefore, the dispersion of Δ*Fgrdrp2* ascospores may signify premature dissolution of the ascus wall. In Δ*Fgrdrp2*, almost all ex-siRNAs were downregulated or absent (Fig. 7C). Previous studies indicated that ex-siRNAs affected ascospore development and discharge in Δ*Fgdcl1/2* by negatively regulating corresponding genes of ex-siRNAs (15). Similarly, we also found the negatively relationship between the ex-siRNAs and the corresponding genes (Fig. 8A and B), and certain corresponding genes of downregulated ex-siRNAs in Δ*Fgrdrp2* were associated with the development or discharge of ascospores. Combined with the results of ascospore discharge in the deletion mutants, we suggested that these downregulated ex-siRNAs may also contribute to the defective ascospore discharge of Δ*Fgrdrp2*.

*Fgrdrp2* and *Fgrdrp3* were significantly induced during sexual reproduction, whereas Δ*Fgrdrp3* did not differ significantly from wild-type PH-1 in perithecium formation, ascospore development, and ascospore discharge. When *Fgrdrp2* and *Fgrdrp3* were knocked out simultaneously, ascospore discharge was not blocked and was indistinguishable from Δ*Fgrdrp2*. To our knowledge, the homolog of *Fgrdrp3*, *N. crassa* RRP-3 with unknown biological function, was not involved in the quelling or MSUD pathway (75). Hence, we speculated that *Fgrdrp3* may not participate in the *rdrp2*-dependent RNAi pathway in sexual reproduction.

When *Fgrdrp5* was deleted, the reduction of ascospore discharge was second only to Δ*Fgrdrp2* (Fig. 3E). Immature ascospores with two nuclei, lower turgor pressure, and reduced expression of genes related to lipid metabolism and ABC transporters may be responsible for the reduced ascospore discharge (Fig. 9C). Moreover, certain unique downregulated DEGs of Δ*Fgrdrp5* were significantly enriched in ribosome biogenesis (Fig. 3). *Fgrdrp5* contains a DEAD box in addition to the RdRP domain. The protein interaction network (*P* value: 9.47e−05) also reflected that *Fgrdrp5* may be associated with ATP-dependent RNA helicase DBP4, ATP-dependent helicase NAM7, and nonsense-mediated mRNA decay factor (Fig. S4). As an RNA-dependent ATPase or RNA helicase, DEAD box genes are involved in pre-mRNA splicing or rRNA assembly, respectively. *RRP3* (rRNA processing), a DEAD box protein that has a weak RNA-dependent ATPase activity and acts as an RNA helicase, is required for the processing of 18S rRNA in *Saccharomyces cerevisiae* (76). Thus, we infer that the DEAD domain of *Fgrdrp5* may also exhibit RNA helicase activity and be involved in rRNA processing, but the mechanism and whether it affects sexual development need to be further confirmed. In *Saccharomyces cerevisiae*, NAM7 has enhanced ATP hydrolytic activity, RNA helicase activity, and ribosomal small subunit binding activity and is homologous to human UPF1, an RNA-dependent helicase required for NMD of abnormal mRNAs containing premature termination codon, and regulated the expression level of normal mRNA (77, 78). The pre-mRNA or NMD pathways during sexual reproduction need to be further explored, and whether these pathways are regulated by *Fgrdrp5* needs to be further confirmed.

## Data Availability

Raw data of RNA-seq and sRNA-seq of Fgrdrp mutants have been deposited in NCBI’s Sequence Read Archive (BioProject: PRJNA884510 and PRJNA888203; SRA metadata: PRJNA884510 and PRJNA888203).

## SUPPLEMENTAL MATERIAL

Supplemental figures mbio.00377-24-s0001.docx Fig. S1 to S4.

Table S1 mbio.00377-24-s0002.xlsx PCR primers.

Table S2 mbio.00377-24-s0003.xlsx Phenotypes of *Fgrdrp* deletion mutants.

Table S3 mbio.00377-24-s0004.xlsx Summary statistics of mapped sRNAs.

Table S4 mbio.00377-24-s0005.xlsx Annotations of mapped sRNAs.

Table S5 mbio.00377-24-s0006.xlsx Statistics of clean and mapped reads of RNA-seq.

Table S6 mbio.00377-24-s0007.xlsx Expression pattern of sexual-development-related genes in mutant DEGs.

ASM does not own the copyrights to Supplemental Material that may be linked to, or accessed through, an article. The authors have granted ASM a non-exclusive, world-wide license to publish the Supplemental Material files. Please contact the corresponding author directly for reuse.
