# Supplementary material for: RNA-dependent RNA polymerases regulate ascospore discharge through the exonic-sRNA-mediated RNAi pathway
Source: mBio. 2024 May 16;15(6):e00377-24. doi: 10.1128/mbio.00377-24 (PMC11237814; doi:10.1128/mbio.00377-24)
Supplement: Supplemental figures — Fig. S1 to S4. [file mbio.00377-24-s0001.docx]

**Supplementary Figures**

**
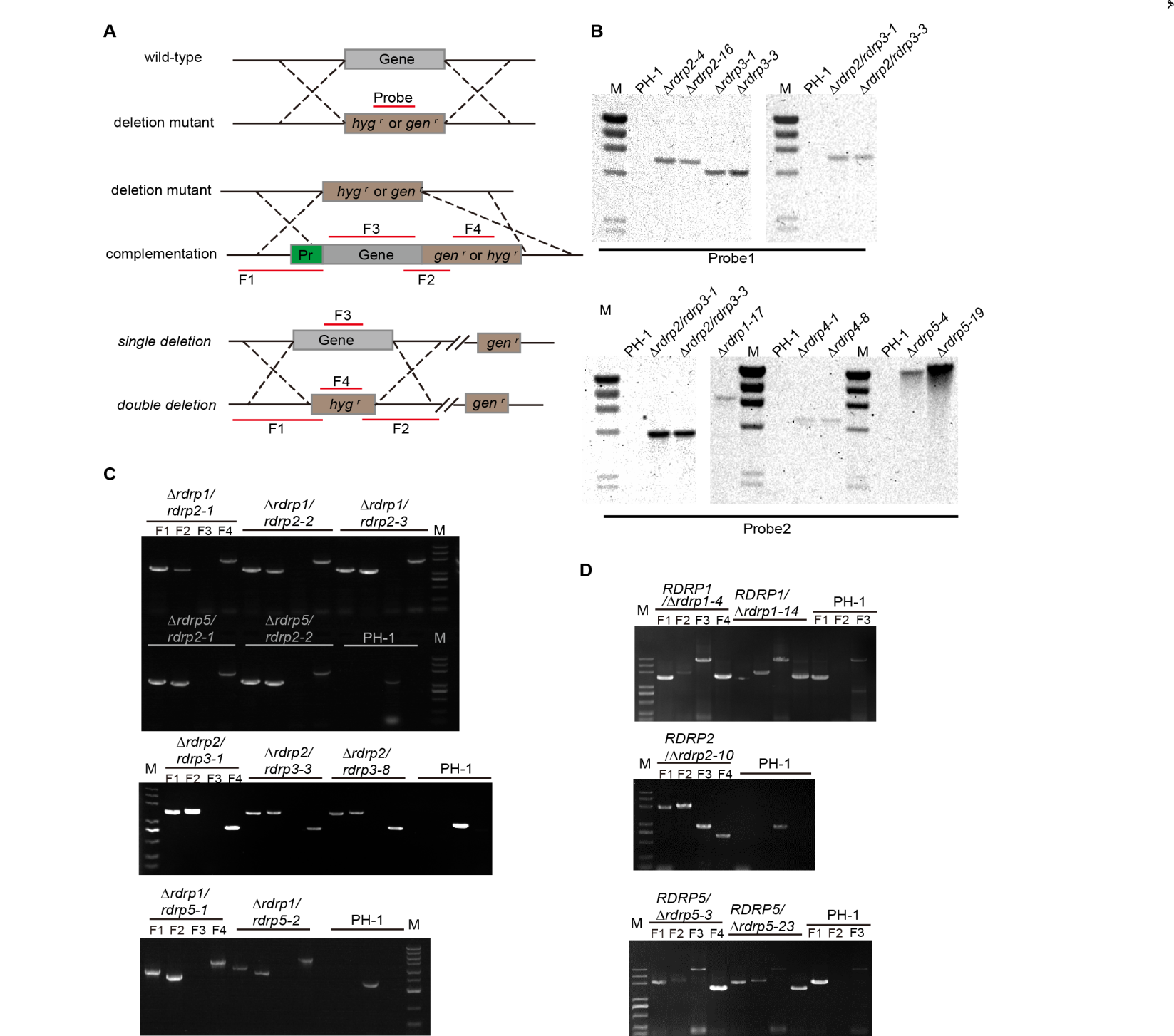
**

**FIG S1** Verification of deletion mutants using Southern blotting and PCR**.** **(A)** Construction strategies for gene deletion and complementation. Red lines indicate the PCR amplified fragment for the deletion mutant identification. gen^r^, geneticin resistance gene; hyg^r^, hygromycin resistance gene. **(B)** Southern blotting analysis. Probe 1 and probe 2 were amplified from hygromycin B gene and geneticin gene, respectively. **(C)** Verification of double-deletion mutants by PCR amplification. *ΔFgrdrp1/2* and *ΔFgrdrp5/2* mutants were generated by transforming protoplasts of *ΔFgrdrp1* and *ΔFgrdrp5,* respectively, with the fusion deletion fragments of *Fgrdrp2* gene. *ΔFgrdrp1/5* was generated by transforming protoplasts of *ΔFgrdrp1* with the fusion deletion fragments of *Fgrdrp5.* *ΔFgrdrp2/3* was generated by transforming protoplasts of *ΔFgrdrp2* with the fusion deletion fragments of *Fgrdrp3.* **(D)** Verification of complementation strains. F1, F2, F3, F4 indicate the fragment F1, F2, F3, and F4, respectively. M, marker.


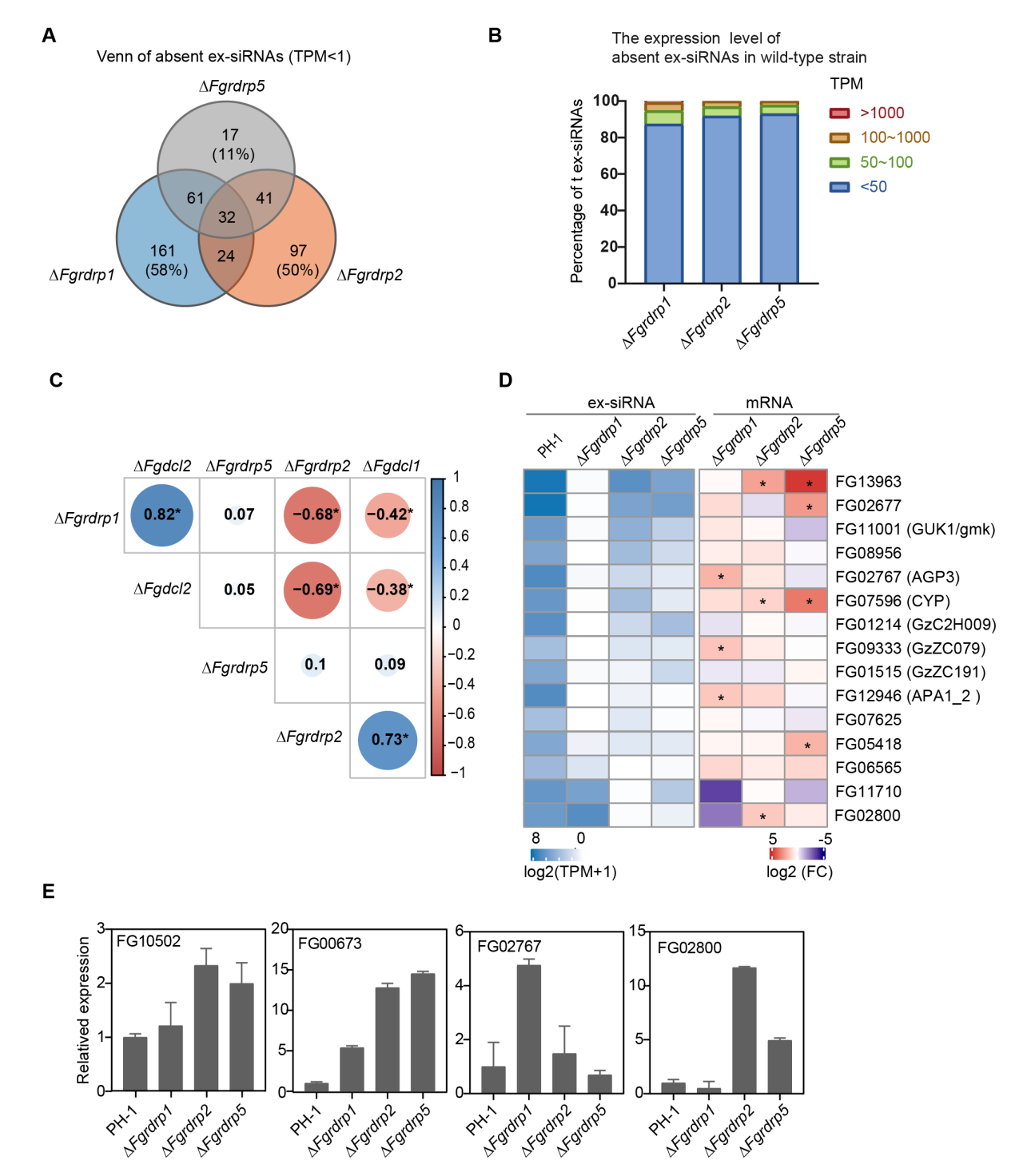


**FIG S2** Characterization of absent ex-siRNAs and ex-siRNA corresponding genes. **(A)** Venn of absent ex-siRNAs. **(B)** Categorical statistics on the expression levels of the absent ex-siRNAs in the wild-type strain. **(C)** The correlation of ex-siRNAs between *Fgdcl* and *Fgrdrp* mutants. The correlation analysis was performed by Pearson’s coefficient depending on the log2 value of the fold change between mutants and the wild-type strain. The TPM values of the absent ex-siRNA were replaced with 1*10^-2^. * indicates p $\leq$ 0.05. **(D)** The expression patterns of ex-siRNAs and corresponding genes cause sexual defects**.** The asterisk indicates the absolute value of log2(FC) is greater than 1. **(E)** Relative expression of ex-siRNAs corresponding genes in *Fgrdrp*-deletion mutants and the wild-type strain PH-1. The total RNAs were isolated from the 7-day-old perithecia and the expression levels of gene were determined by RT-qPCR. The expression levels of these corresponding genes in PH-1 were used as a control (set to 1). Line bars indicate standard deviation from three repeated experiments. Genes FG10502 and FG00673 are the corresponding genes of ex-sR004 and ex-sR005 (shown in Fig7D), respectively.

**
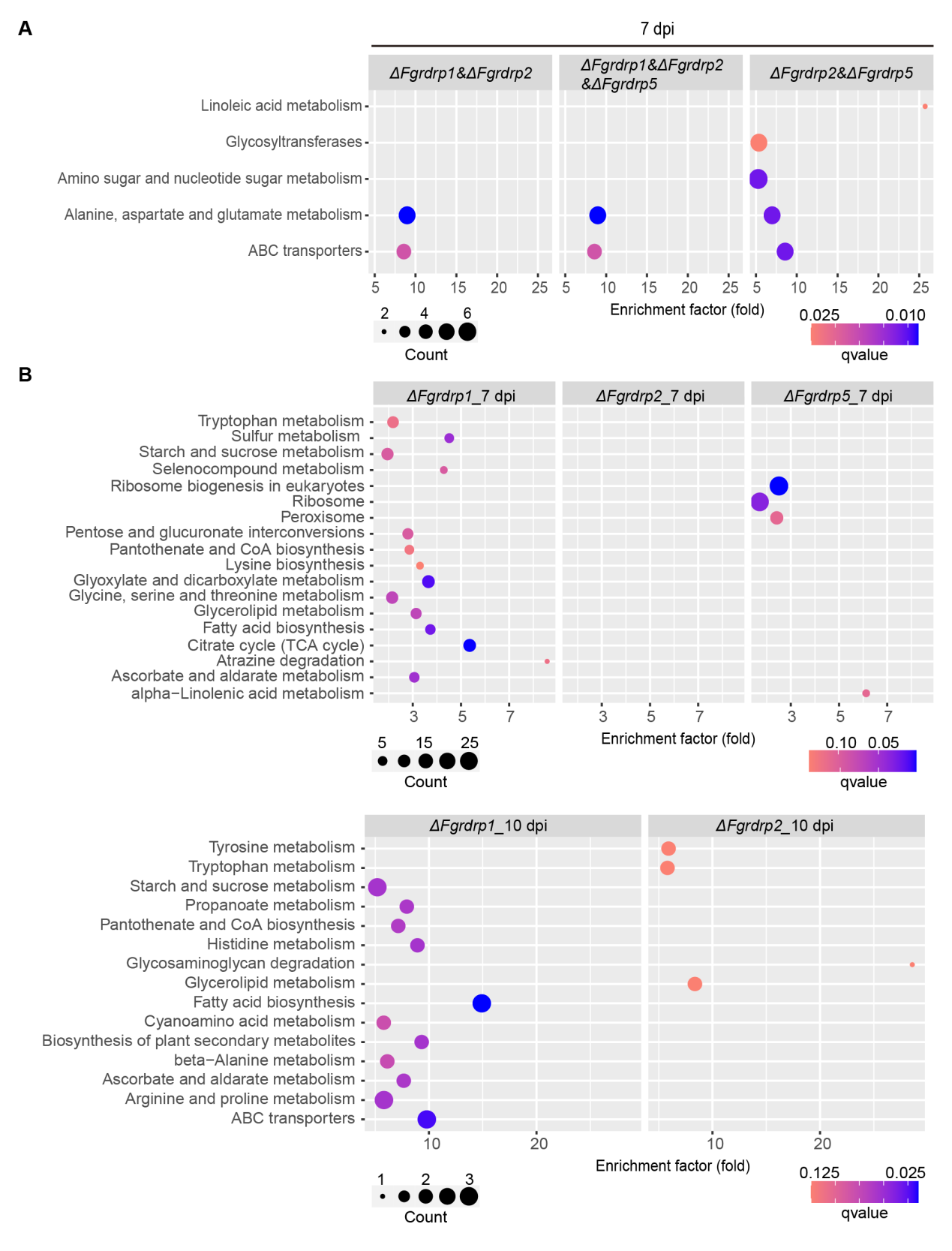
**

**FIG S3** The KEGG pathways enriched by DEGs of *Fgrdrp*-deletion mutants. **(A)** Enriched KEGG pathways in the common DEGs of *Fgrdrp*-deletion mutants. **(B)** Mutant-specific KEGG pathway enriched by DEGs.

**
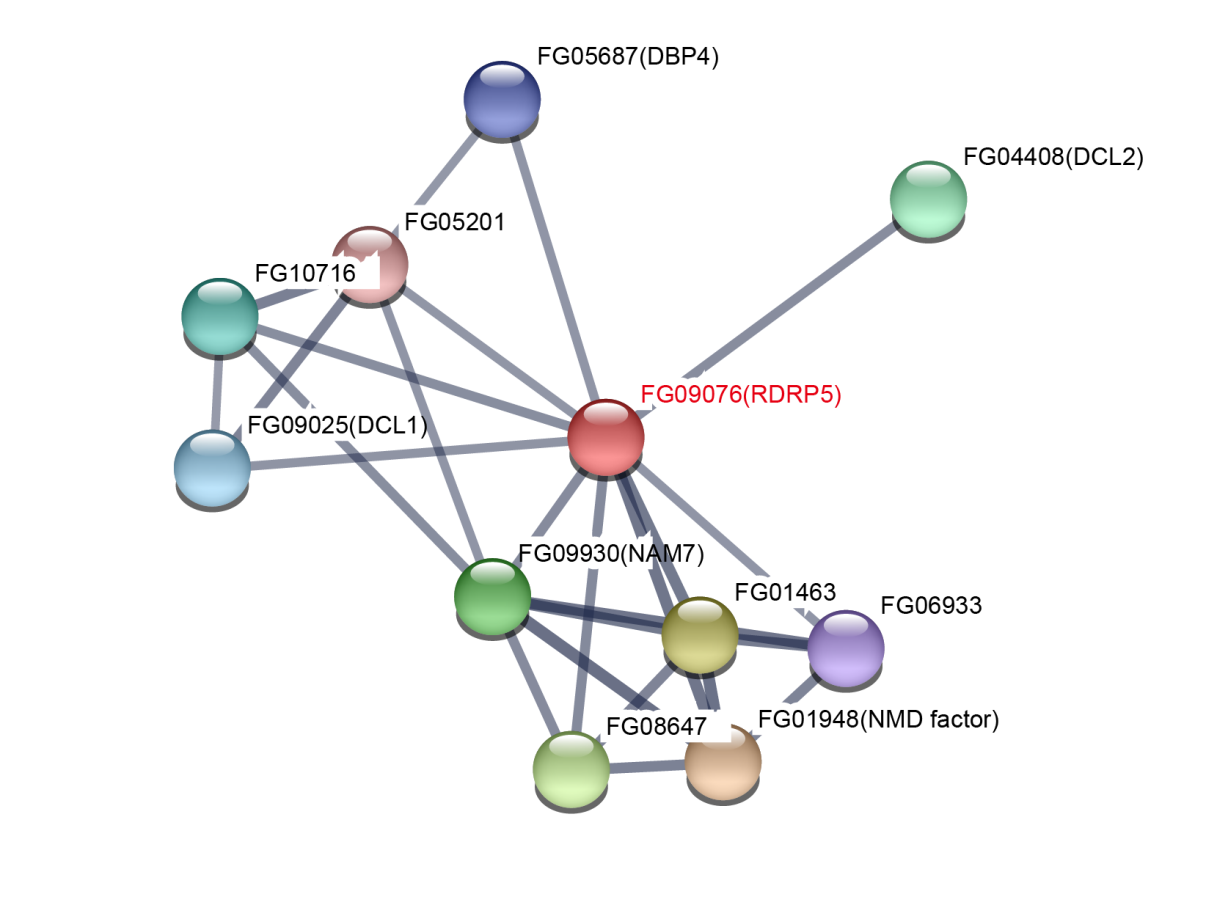
FIG S4** Protein-protein interaction (PPI) network of *FgRdRP5*. Protein-protein interaction (PPI) network of *FgRdRP5*. The interaction network was predicted by STRING 11.5. The minimum required interaction score was 0.7, and the interaction sources included text, experiments, databases, co-expression and co-occurrence. Network nodes represent proteins, line thicknesses indicate the interaction score. NAM7, ATP-dependent helicase NAM7; NMD factor, a hypothetical protein similar to nonsense-mediated mRNA decay factor; DBP4, ATP-dependent RNA helicase DBP4, ATP-dependent RNA helicase required for ribosome biogenesis.
